# Graph-Based Machine Learning for Predicting Drug–Drug Interactions: A Systematic Review

**DOI:** 10.3390/ph19081225

**Published:** 2026-08-04

**Authors:** Md. Tuhin Reza, Md. Abdul Kader, Wissem Inoubli, Md. Biddut Hossain, Md. Kamrul Islam

**Affiliations:** 1 Department of Computer Science & Engineering, Jashore University of Science & Technology, Jashore 7408, Bangladesh; tuhin.reza@fec.ac.bd; 2 Department of Software Engineering, Daffodil International University, Daffodil Smart City, Dhaka 1216, Bangladesh; abdulkader.swe@diu.edu.bd; 3 Computer Science, University of Artois, 62000 Arras, France; wissem.inoubli@univ-artois.fr; 4 ICT Division, Bangladesh Bureau of Educational Information & Statistics (BANBEIS), Dhaka 1207, Bangladesh

**Keywords:** drug–drug interaction, graph neural network, computational pharmacology, multimodal learning, attention mechanism

## Abstract

**Background/Objectives:** Drug–drug interactions (DDIs) are major medication-safety concerns, and experimental testing cannot cover the expanding number of drug pairs. This systematic review evaluates graph-based machine-learning methods for DDI prediction, focusing on machine-learning architectures, data integration, interpretability, reproducibility, and clinical relevance. **Methods:** Following PRISMA 2020, we systematically searched major databases for studies published between January 2021 and March 2026. We included studies that applied graph-based machine-learning models, particularly graph neural networks, to predict DDIs. We compared their data sources, model designs, validation methods, predictive performance, reproducibility, and clinical relevance. Because the studies used different datasets and evaluation methods, the findings were summarized narratively rather than combined statistically. **Results:** Sixty studies met the eligibility criteria. Methods progressed from graph convolutional networks and graph attention networks to graph transformers, contrastive learning, multimodal fusion, and LLM-enhanced representations. We found that reported improvements in prediction performance often remained study-specific. Only three studies explicitly mentioned or addressed data leakage, whereas most reviewed studies contained no explicit leakage discussion; leakage-aware drug-disjoint, temporal, and external evaluations were also uncommon. Uncertainty calibration, computational-resource reporting, complete reproducibility materials, and independently validated explanations were also limited. **Conclusions:** Graph-based machine learning is promising for DDI prioritization and hypothesis generation but remains insufficient for independent clinical decision-making. Future studies should use standardized benchmarks, leakage-aware validation, calibrated uncertainty, reproducible pipelines, validated explanations, and external or prospective evaluation.

## 1. Introduction

Drug–drug interactions (DDIs) occur when the effect or safety profile of one drug is altered by another drug. These interactions can reduce therapeutic efficacy, increase toxicity, or contribute to preventable adverse drug events, especially in patients receiving multiple medications [1,2,3]. Clinical pharmacology studies, post-marketing surveillance, and expert-curated resources remain well-established approaches for identifying clinically important DDIs, while laboratory experiments provide proactive evidence for assessing interaction risk. However, exhaustive experimental screening is not feasible because the number of possible drug pairs expands rapidly as new drugs, indications, and combination therapies enter practice [4,5]. Computational DDI prediction is therefore an important complementary strategy for prioritizing risky combinations, guiding drug-development decisions, and supporting medication-safety workflows.

Early computational DDI methods relied mainly on manually engineered chemical descriptors, similarity measures, side-effect profiles, target annotations, or interaction-network features. These approaches demonstrated that heterogeneous biomedical evidence can improve prediction, but they often struggled to represent complex dependencies among drugs, proteins, enzymes, transporters, pathways, diseases, phenotypes, and adverse events [5,6,7]. Recent deep-learning (neural network-based) architectures have reframed DDI prediction as a representation-learning problem in which drugs and biomedical entities are embedded from molecular structures, DDI networks, knowledge graphs, biomedical text, and multimodal combinations of these sources [8,9]. This significant shift is particularly relevant because DDIs are inherently relational; the risk associated with a drug pair depends not only on the properties of the individual molecules but also on the biological and clinical context in which they interact.

Recently, graph neural networks (GNNs) have gained increasing attention for DDI prediction due to their ability to capture complex topological relationships within drug interaction networks. In DDI graphs, biomedical entities (e.g., drugs, proteins, genes, enzymes, diseases, pathways, side effects) are represented as nodes (or vertices), and known interactions between pairs of entities are represented as edges (or links). Hypergraphs and graph transformers further extend GNNs by modeling higher-order relations and long-range dependencies [10,11,12,13,14,15]. Recent studies also combine GNNs with attention mechanisms, contrastive learning, functional-group-aware representations, large language model (LLM)-derived features, 2D/3D molecular encoders, and multimodal fusion [16,17,18,19,20,21]. As a result, GNN-based DDI prediction is no longer a single model type, but a broad design space involving graph construction, modality choice, attention, fusion, supervision, validation, and interpretability. A central question for researchers and clinicians is whether GNN-based DDI predictions are valid and accurate. For this review, internal validity refers to performance on the benchmark dataset and split used for development, whereas external or translational validity refers to generalization beyond those conditions [8,9,22]. Most included studies assessed internal validity using cross-validation or held-out benchmark sets and reported AUROC, AUPR, F1 score, or accuracy. Representative study-specific results include 94.47% transductive accuracy for SSI-DDI on a DrugBank event benchmark, 99.74% warm-start accuracy for DrugDAGT, and 95.43% AUROC for KnowDDI on TWOSIDES [11,23,24]. Because datasets, tasks, and splits differ, these results are not directly comparable and do not by themselves establish external or clinical validity.

The principal threats to validity identified in this review are (1) leakage-prone random pairwise splits, in which the same drug identities or related evidence can occur in training and test sets [9,22]; (2) incomplete or noisy labels, particularly when unobserved interactions are treated as confirmed negatives [5,6]; (3) limited cold-start evaluation on genuinely unseen drugs [25,26]; and (4) scarce temporal evaluation [9,22]. DSIL-DDI and DSN-DDI reported unseen-drug evaluations [25,26]; KnowDDI and SGT-DDI provided explanatory graph paths or attention-based evidence [17,24]; and KA-DDI and CASE used contrastive objectives to improve robustness in sparse or imbalanced settings [20,27]. Such efforts remain exceptions. The present review therefore considers predictive accuracy together with validation rigor, label quality, reproducibility, interpretability, and clinical applicability.

Several reviews have examined traditional machine-learning and deep-learning approaches, including methodologies, biomedical databases, explainability, efficiency, and cold-start evaluation [5,6,8,9,22,28]. A focused synthesis is nevertheless needed to connect graph construction, architecture, attention, fusion, and validation in recent graph-based models.

Attention mechanisms, multimodal fusion, and graph architecture choices are now central to many recent DDI-prediction models. Attention can weight atoms, molecular fragments, neighboring drugs, biomedical relations, or input modalities [11,16,29,30]. Multimodal fusion combines molecular, network, biological, and textual evidence to represent broader pharmacological context [17,31,32,33]. These developments motivate examining when such components add measurable and interpretable value beyond simpler designs.

This review therefore synthesizes how graph construction, GNN architecture, attention, multimodal fusion, and validation interact. It emphasizes (i) graph datasets and biomedical data sources; (ii) message-passing GNNs, graph transformers, hypergraphs, and contrastive frameworks; (iii) attention and fusion strategies; (iv) benchmark design, reproducibility, and risk of bias; and (v) interpretability and translation.

As a systematic review rather than a primary experiment, it is organized around three analytical questions: (RQ1) how graph construction, data modality, and validation design influence reported performance; (RQ2) when attention and multimodal fusion add demonstrable value beyond simpler baselines; and (RQ3) what evidence supports translation beyond benchmark performance. These questions guide a qualitative synthesis rather than formal hypothesis testing.

### 1.1. Preliminaries

Computational DDI prediction transforms pharmacological evidence into machine-readable representations and uses a model to learn a label for each drug pair $\left(d_{i},d_{j}\right)$, such as interaction/no interaction, event type, mechanism category, severity, or cold-start risk for unseen drugs [5,6,8,9]. In the reviewed studies, drugs are represented using molecular graphs, SMILES sequences, descriptor vectors, target or side-effect profiles, text embeddings, DDI-network neighborhoods, or biomedical knowledge-graph contexts. Graphs therefore encode atoms and bonds, drugs and known interactions, or heterogeneous entities such as drugs, proteins, enzymes, diseases, pathways, and adverse events.

Three modeling concepts recur throughout this review:

1. GNN architectures refer to graph-learning designs used to represent and update drug or biomedical-entity embeddings, including message-passing GNNs, graph attention networks, graph transformers, hypergraph models, and contrastive graph frameworks.
2. Multimodal fusion means combining more than one type of drug information, such as molecular structure, SMILES, DDI networks, knowledge graphs, side effects, targets, pathways, or biomedical text. Component ablation is needed to determine whether added modalities contribute useful information or introduce noise, missing-data bias, or redundancy [17,31,32,33].
3. Attention mechanisms allow models to weight different parts of the input more strongly, such as key atoms, molecular fragments, neighboring drugs, knowledge-graph relations, or data sources. Attention can help identify which evidence the model emphasizes, but it should not be treated as definitive proof of a biological mechanism without further validation [11,16,29,30].

### 1.2. General Workflow for DDI Prediction

**Figure 1:**
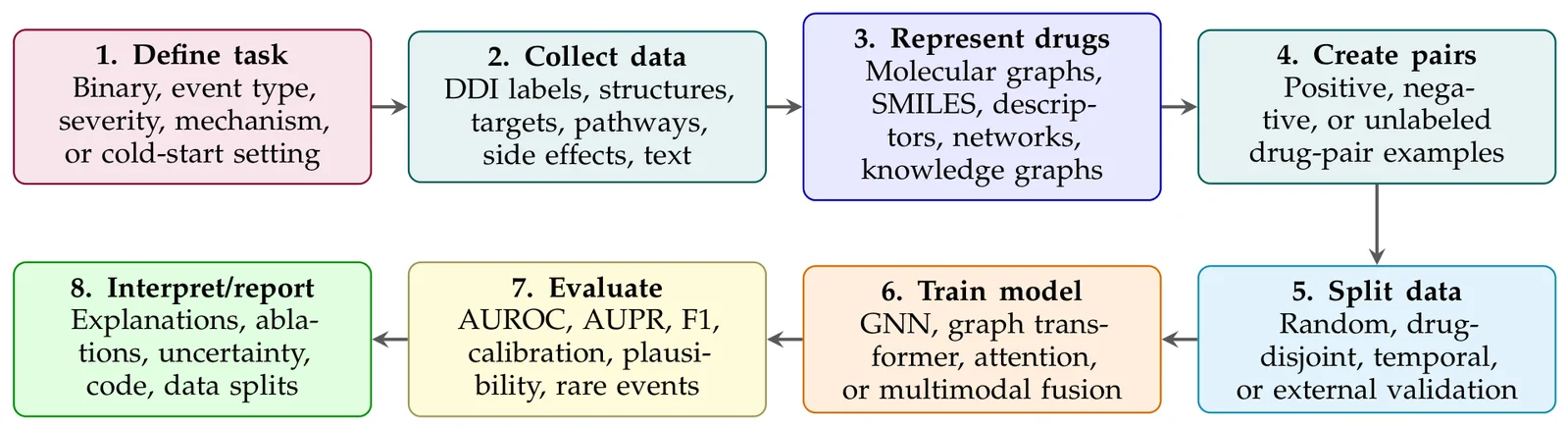
General workflow for computational DDI prediction, linking pharmacological problem formulation with machine-learning data preparation, modeling, evaluation, interpretation, and reproducible reporting.

**Figure 2:**
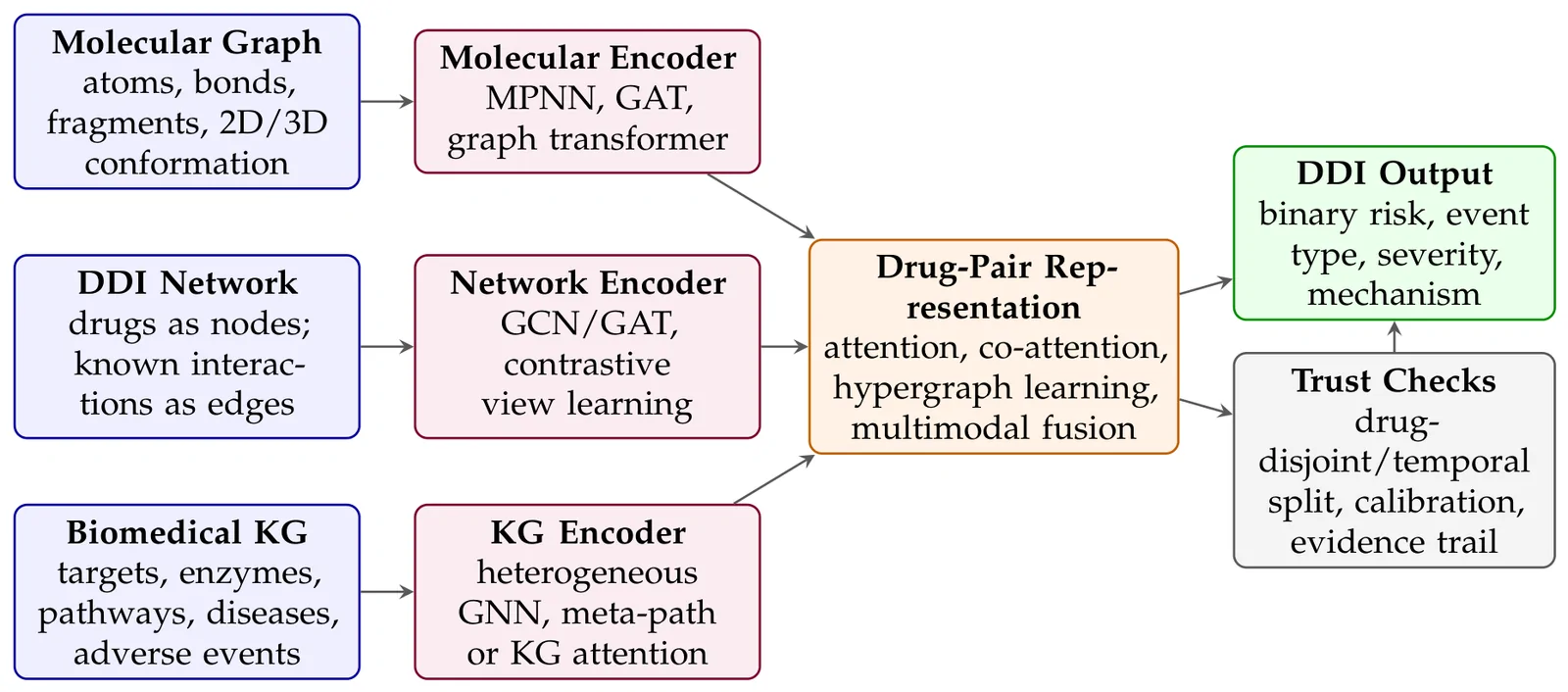
Domain-specific graph-learning architecture for computational drug–drug interaction (DDI) prediction.

A general DDI prediction workflow begins by defining the task, matching drug identifiers with labels, building molecular or biomedical representations, creating drug pairs, and selecting appropriate data splits. Researchers then train a GNN, graph transformer, hypergraph, contrastive, attention-based, or multimodal model and evaluate it with task-appropriate metrics, robustness tests, ablations, and explanatory evidence [28,34,35]. Figure 1 summarizes this workflow, and Figure 2 shows a graph-learning architecture for DDI prediction.

DDI prediction studies commonly use DrugBank, TWOSIDES, SIDER, KEGG, PharmGKB, and biomedical knowledge graphs linking drugs with targets, enzymes, diseases, pathways, and adverse-event phenotypes. Label provenance, negative-sample construction, and overlapping evidence can substantially influence reported performance [5,6,8,9]. Supplementary Table S2 summarizes the datasets and reported performance values across the 60 reviewed studies.

The workflow highlights the need to align the pharmacological question, graph representation, model architecture, fusion strategy, validation protocol, and interpretability evidence. The following Section 2 describes how studies were identified and synthesized across these dimensions.

## 2. Review Methodology

### 2.1. PRISMA Framework, Search Strategy, and Selection

**Figure 3:**
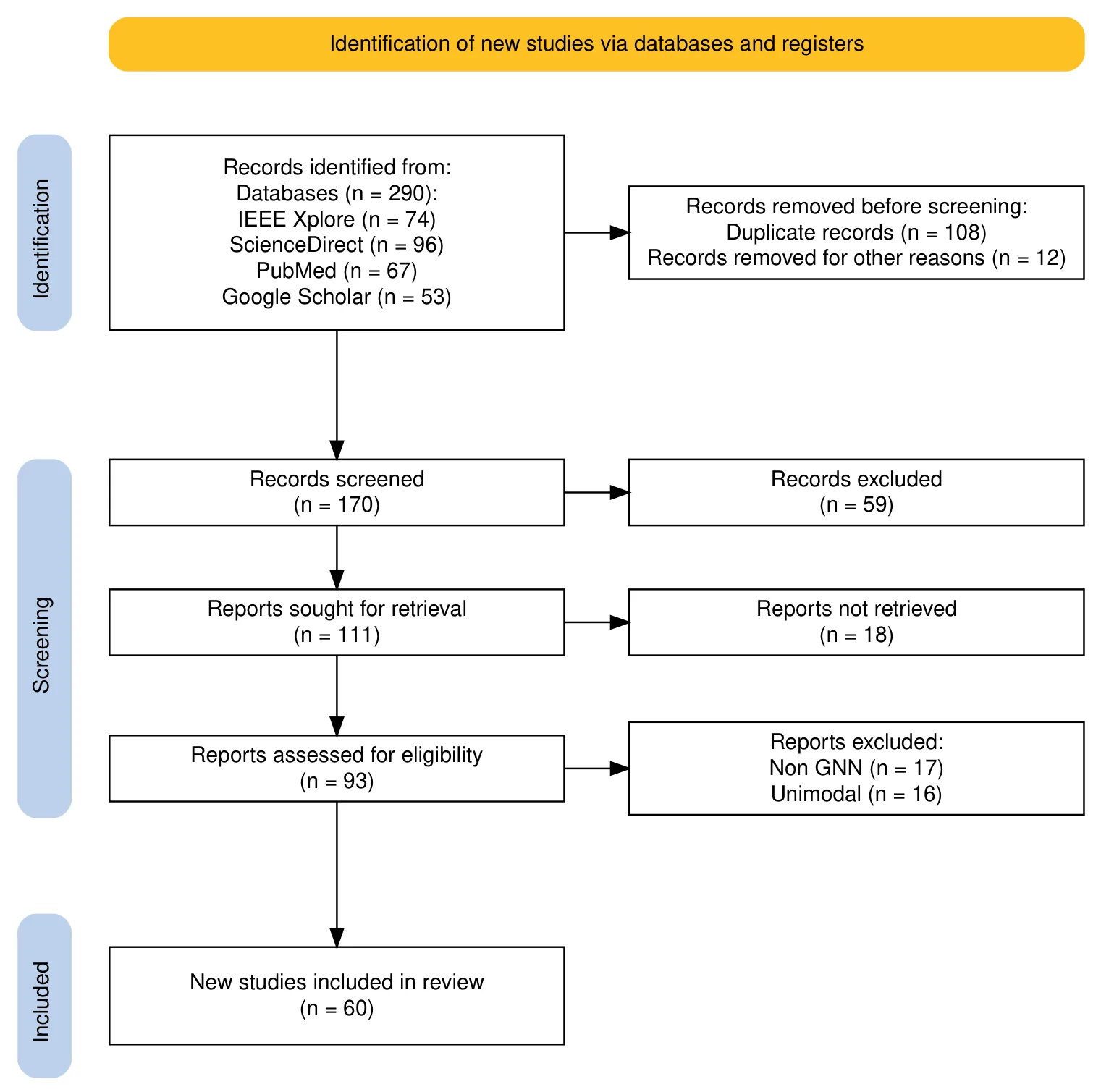
Completed PRISMA 2020 flow diagram for identification, screening, eligibility assessment, and inclusion of studies in the review.

This systematic review was designed, conducted, and reported in accordance with the PRISMA 2020 statement and checklist for transparent identification, screening, eligibility assessment, and inclusion of evidence [34,35]. A completed PRISMA 2020 flow diagram is provided in Figure 3, and a completed PRISMA 2020 checklist mapping each item to the manuscript location is provided in Supplementary Table S1. Because the aim was methodological synthesis rather than clinical-effect estimation, the review was not prospectively registered in PROSPERO or another registry, and no separate protocol document was published. No protocol amendments were made. This review examined how GNNs and graph-enhanced deep-learning models have been used for computational DDI prediction. It also considered how graph representation, modality choice, architecture, validation, and interpretability affect reliability. Owing to heterogeneity in datasets, labels, negative sampling, validation splits, and metrics, meta-analysis was not performed.

Searches covered peer-reviewed journal and conference papers on GNN-based or graph-enhanced DDI prediction published during 2021–2026 (through 15 March 2026), with foundational, review, and clearly identified contextual papers retained outside the primary evidence count. The final search update was conducted on 15 March 2026 for IEEE Xplore, ScienceDirect, PubMed, and Google Scholar. Searches used DDI, graph neural network, knowledge graph, hypergraph, contrastive learning, multimodal fusion, and molecular-graph terms; Boolean strings were adapted to each database interface, and the applied filters and record counts are reported in Table 1. For Google Scholar, the first 100 relevance-ranked results were examined; 53 records remained for the identification set after applying the stated date, document-type, and scope filters. Backward and forward citation tracing was used to verify coverage and identify contextual sources [5,6,8,9]. No trial registers, regulatory registers, organizational websites, or clinical-study repositories were searched because the review question targeted computational-method papers rather than clinical intervention studies. The search identified 290 database records: IEEE Xplore (n = 74), ScienceDirect (n = 96), PubMed (n = 67), and Google Scholar (n = 53). After removing 108 duplicates, 12 additional records were removed during pre-screening because their full bibliographic metadata showed that they failed the language, document-type, date, or computational-DDI scope criteria. The remaining 170 titles and abstracts were screened; 111 reports were sought for full-text review, 93 were assessed for eligibility, and 60 primary studies were included. Reports not retrieved (n = 18) were inaccessible full texts, duplicate conference/journal versions for which the fuller version was retained, or records for which only insufficient bibliographic information was available. Supplementary Table S4 provides the complete aggregate screening accounting, and Figure 3 summarizes selection.

### 2.2. Eligibility Criteria

Studies were eligible when they met all of the following criteria: (i) the task involved prediction, event classification, graph-supported extraction, or prioritization of DDIs; (ii) the method used a GNN, graph-derived neural representation, biomedical knowledge graph, molecular graph, DDI network, hypergraph, or graph-enhanced multimodal model; (iii) the study reported empirical evaluation using benchmark datasets, curated biomedical resources, or clinically relevant DDI labels; and (iv) sufficient methodological detail was available to identify data sources, graph construction, model architecture, and evaluation metrics. Within this review, DDI prediction is used as an umbrella term that includes graph-based event classification and graph-supported DDI extraction, while these task types remain distinguished in the evidence tables. To avoid scope creep, we distinguished graph-based models, where a molecular graph, DDI network, knowledge graph, or hypergraph is the primary relational backbone used for message passing or graph reasoning, from graph-enhanced models, where graph-derived features or KG paths are one input modality within a broader multimodal or LLM-enhanced pipeline. Graph-enhanced studies were retained only when the graph component contributed directly to the DDI pipeline and was identifiable in the architecture or reported inputs. Studies were excluded when they focused only on non-DDI drug discovery tasks, lacked empirical evaluation, used only traditional non-graph machine-learning methods, were unimodal without a graph component, or provided insufficient methodological detail. Titles and abstracts were screened against the eligibility criteria by at least two members of the author team, and each retrieved full-text report was assessed by at least two authors. Screening was performed in parallel rather than blinded; disagreements were resolved by discussion and consensus with the full author team when needed. No automated eligibility-decision tools were used. During full-text eligibility assessment, 17 reports were excluded because they did not use GNN or graph-based neural methods, and 16 were excluded because they were unimodal or otherwise outside the focused graph-enhanced synthesis scope. No full-text report that otherwise met the graph-based or graph-enhanced DDI eligibility criteria was excluded solely because of low benchmark performance or unfavorable results. The final PRISMA-included evidence base therefore comprised 60 primary graph-based or graph-enhanced DDI studies. Additional foundational, methodological, pharmacological, reporting-guideline, and web resource references were cited for context but were not included in the primary evidence synthesis.

### 2.3. Data Collection Process and Data Items

For each included study, data were extracted using a structured evidence matrix and cross-checked within the author team. At least two authors collected or verified data from each included report; extraction was not blinded, and disagreements were resolved by consensus. No automated data-extraction tools were used, and study investigators were not contacted because all synthesis variables were taken from published reports. Extracted items included publication year, prediction task, graph type, node and edge definitions, biomedical data sources, input modalities, GNN architecture, message-passing strategy, attention mechanism, fusion mechanism, benchmark dataset, validation design, negative-sampling procedure, reported performance metrics, ablation results, interpretability method, code or data availability, funding/support statements where reported, and stated limitations. Supplementary Table S3 provides the operational eligibility, extraction, and decision rules. When a study reported multiple experiments, emphasis was placed on the primary DDI prediction task and the evaluation setting most relevant to generalization. All reported DDI-prediction outcomes for the primary model and main comparators were sought, with AUROC, AUPR, F1 score, accuracy, precision, recall, and related benchmark metrics extracted when available. Missing or unclear information was recorded as not reported rather than imputed. No clinical effect measures were defined because this review synthesized computational model performance rather than intervention effects; benchmark metrics were therefore extracted as reported by the original studies, and no metric transformations were applied.

### 2.4. Risk-of-Bias and Applicability Assessment

Risk of bias and applicability were assessed using a structured domain-based framework rather than relying only on narrative judgment. At least two authors assessed each included study; assessments were not blinded, and disagreements were resolved by consensus. No automated risk-of-bias tools were used. Overall concern was judged with reference to seven domains: data leakage, negative sampling, generalization design, baseline and ablation adequacy, reproducibility, interpretability, and clinical applicability. Because reporting was incomplete and the reviewed tasks were heterogeneous, the domains were not converted into a numerical score or presented as falsely precise per-domain ratings. Instead, Table 2 defines the assessment criteria and Table 3 reports a transparent overall grouping with the dominant reasons for concern. The study-level evidence map in Supplementary Table S2 and the narrative synthesis provide the supporting task, dataset, validation, reproducibility, and interpretation context. Formal statistical assessment of publication bias or small-study effects was not appropriate because no pooled effect estimate or meta-analysis was performed; possible reporting bias was therefore considered qualitatively. Certainty of evidence was not graded with GRADE because the review did not evaluate intervention effects.

### 2.5. Synthesis Methods

Because the included studies varied substantially in datasets, graph construction, task definitions, negative sampling, validation protocols, and reported metrics, quantitative meta-analysis was not appropriate. Findings were synthesized narratively and through structured tables, consistent with systematic-review guidance for heterogeneous evidence bases [34,35,80]. Studies were eligible for each thematic synthesis when they reported enough detail about the relevant dimension, such as architecture, attention mechanism, multimodal fusion, validation protocol, interpretability, or scalability. Reported improvements such as percentage-point gains in F1, accuracy, or AUROC were treated as within-study findings from the original authors, not as pooled or directly comparable cross-study effect sizes. Models were grouped into architecture families, including molecular-graph GNNs, DDI-network models, heterogeneous knowledge-graph models, hypergraph neural networks, graph-transformer methods, contrastive-learning approaches, attention-based models, and LLM-enhanced multimodal systems. Data were prepared for synthesis by harmonizing terminology for model families, graph types, modalities, and evaluation protocols while preserving the original reported metrics and study claims. Tabular syntheses summarize search results, bias domains, model-family trade-offs, validation protocols, modality checks, study-level datasets and reported values, and clinical-translation pathways. Heterogeneity was explored narratively by comparing dataset source, graph construction, validation split, negative sampling, metric choice, ablation reporting, and external or temporal evaluation. No subgroup meta-analysis, sensitivity meta-analysis, or certainty-weighted pooling was conducted because the studies did not share compatible effect measures or standardized data partitions.

### 2.6. Completed PRISMA 2020 Checklist

The completed PRISMA 2020 checklist and the manuscript location for each reporting item are provided in Supplementary Table S1.

## 3. Results

This section synthesizes how graph construction, architecture, attention, fusion, and validation influence DDI prediction. Recent studies increasingly use knowledge graphs, graph transformers, hypergraphs, contrastive learning, LLM-derived features, and multimodal fusion, but reported gains remain dataset- and protocol-specific. The analysis therefore emphasizes methodological trade-offs and representative evidence rather than paper-by-paper performance rankings.

### 3.1. Critical Synthesis Across Model Families

Model families differ mainly in the evidence they encode and the generalization setting they support. Molecular graphs emphasize chemistry, DDI networks emphasize interaction topology, knowledge graphs add mechanistic context, and hypergraph or multimodal models represent higher-order or complementary evidence. Table 4 summarizes the resulting strengths, limitations, and validation requirements.

Cross-study metrics should not be compared without considering label construction, negative sampling, and split design; these issues are examined in Section 4.9.

### 3.2. Graph Neural Network Architectures in DDI Prediction

The reviewed architectures fall into basic message-passing, dual-view or multiscale, heterogeneous knowledge-graph, transformer, hypergraph, contrastive/self-supervised, and architecture-search families. Representative examples illustrate the progression: SSI-DDI established substructure-aware message passing; DSN-DDI separated intra- and inter-drug views; HetDDI and KnowDDI added biomedical knowledge and inspectable paths; DrugDAGT and SGT-DDI introduced long-range and 2D/3D reasoning; MFHNN-DDI modeled higher-order relations; KA-DDI targeted sparse-label robustness; and AutoDDI explored dataset-specific operator selection [11,13,17,18,20,23,24,26,81].

These examples show that added complexity is useful only when it contributes distinct evidence and is supported by appropriate ablation and generalization tests. The family-level trade-offs are already summarized in Table 4; Section 4 retains only a selective model inventory. The detailed architecture advantages and limitations and the extended inventory of heterogeneous, knowledge-graph, transformer, contrastive, and architecture-search models are provided in Supplementary Tables S6 and S8.

### 3.3. Attention Mechanisms in DDI Prediction

Attention mechanisms appear across molecular-graph, DDI-network, knowledge-graph, transformer, and multimodal DDI models because they provide a learnable way to prioritize atoms, bonds, fragments, neighbors, semantic paths, relation types, or evidence modalities. The reviewed literature can be grouped into seven overlapping attention categories: graph attention, cross-/co-attention, hierarchical attention, knowledge-graph attention, transformer self-attention, feature or modality weighting, and contrastive attention. Because a study may use more than one form of attention, Table 5 integrates the qualitative taxonomy with the principal advantages and limitations of each category rather than presenting mutually exclusive frequency counts.

Graph attention and substructure attention are most useful when local chemical fragments drive the interaction signal, as in SSI-DDI, SA-DDI, DSN-DDI, GMIA, and functional-group-aware models [11,19,26,30,47]. Cross-attention and co-attention explicitly compare two drugs or two modalities and therefore support pair-specific explanations, but their quadratic cost can become limiting for large molecular graphs or many modalities [16,32,82]. Hierarchical and KG attention extend weighting to meta-paths, entity types, and knowledge-graph neighborhoods, improving semantic reasoning when graph relations are reliable [20,74,83,84].

Transformer self-attention expands the receptive field to all nodes or all modality tokens, enabling global dependency capture in graph-transformer and 2D/3D fusion models [17,23,77]. Feature-level attention and attention-weighted fusion learn which modalities or dimensions are most informative, whereas contrastive attention aligns complementary views under weak supervision [12,27,31,85,86]. Across these categories, attention should be treated as an interpretability aid rather than a mechanistic guarantee; credible explanations require ablation studies, chemical-plausibility assessments, pathway-level evidence, and external pharmacological validation.

Attention can improve DDI models by emphasizing relevant substructures, relations, and modalities, but it can also increase computational cost, overfit noisy evidence, or produce visually plausible explanations that lack clinical validation. The merged synthesis in Table 5 makes these trade-offs explicit for each attention category.

**Key Observation:** Attention mechanisms have shifted from local neighbor weighting toward multi-level and cross-modal reasoning. The most convincing uses combine attention with ablation studies and clinically meaningful explanations rather than relying on attention maps alone.

Supplementary Table S9 provides the extended inventory of hierarchical, knowledge-graph, transformer, feature-weighting, and contrastive attention mechanisms removed from the main text for concision.

### 3.4. Multimodal Fusion and Integration Strategies

Multimodal fusion is now central to DDI prediction because no single evidence source fully captures interaction risk. Molecular graphs and SMILES describe chemical structure, DDI networks encode known relational context, knowledge graphs connect drugs to biological mechanisms, and text or LLM-derived representations can add semantic evidence from biomedical literature and labels. The principal integration strategies include simple concatenation, attention-weighted fusion, cross-/co-attention, bi-level or multi-level fusion, masked-autoencoder alignment, hierarchical transformer fusion, orthogonal projection, graph-based integration, LLM-enhanced fusion, and contrastive multimodal learning. These strategies are non-exclusive, and studies that use only a graph backbone should not be interpreted as multimodal solely because they perform graph-based integration.

Simple concatenation and early fusion remain useful baselines because they are transparent and parameter-efficient, but they treat modalities as equally reliable and rarely model cross-modal interactions. Attention-weighted and cross-attention fusion learn which modalities, fragments, or representations matter for a specific drug pair, improving interpretability at the cost of additional complexity [16,23,31,32]. Bi-level, multi-level, graph-based, and hierarchical-transformer fusion models combine local chemical features with global biomedical context, while orthogonal projection and contrastive objectives reduce redundancy and align complementary views [12,17,20,36,87].

LLM-enhanced fusion is an emerging approach for incorporating text-rich DDI evidence. Recent systems use biomedical language models, GPT-derived embeddings, sentence-tree representations, or multimodal extraction to connect unstructured text with structured graphs [21,33,52,88,89]. These methods can improve semantic coverage and zero-shot generalization, but they remain especially vulnerable to rapid model-version changes, prompt sensitivity, retrieval or extraction errors, high-dimensional embeddings, and hidden provenance problems [21,88,90]. For reproducibility, LLM-enhanced DDI studies should report the exact model name and version, access date or checkpoint, prompts, decoding settings, retrieval corpus, extraction rules, and whether generated or extracted text was screened for leakage from benchmark labels [90]. Table 6 integrates the defining characteristics, advantages, and limitations of the principal multimodal-fusion strategies represented in the included studies.

Fusion strategies improve coverage by combining chemical, biological, network, and textual evidence, but they also increase architectural complexity and can conceal leakage or redundant signals. The merged synthesis makes these trade-offs explicit for each integration strategy.

**Key Observations:** Fusion has evolved from feature concatenation toward attention, contrastive alignment, graph-based integration, and LLM-enhanced evidence extraction. The strongest claims come from studies that report modality ablations, missing-modality behavior, cold-start performance, calibration, and external validation rather than benchmark accuracy alone.

Supplementary Table S10 provides the extended inventory of LLM-enhanced, hypergraph, contrastive, and knowledge-adaptive fusion strategies.

### 3.5. Computational Requirements and Scalability

Computational cost and scalability were inconsistently reported across the included studies, limiting deployment-oriented comparisons. Across the 60 included studies, GPU specifications were reported in 8 studies (13.3%), training time in 12 (20.0%), inference time in 6 (10.0%), parameter count in 18 (30.0%), memory use in 8 (13.3%), and scalability analysis in 15 (25.0%). These values describe reporting frequency rather than comparative model efficiency. Table 7 and Supplementary Table S5 provide the corresponding audit, while Section 4.5 discusses the deployment implications of the observed model-family trade-offs.

### 3.6. Integrated Critical Synthesis of GNN Architectures, Attention Mechanisms, and Multimodal Fusion

Architecture, attention, and fusion should be treated as linked design choices rather than independent sources of improvement. The graph defines the evidence structure, attention selects relevant signals, and fusion combines modalities; each component should therefore add distinct information. Chemically driven tasks may need only a molecular GNN, mechanistic tasks may justify heterogeneous knowledge graphs, and unseen-drug settings may benefit from contrastive learning. Across use cases, parsimonious models with matched ablations and drug-disjoint, temporal, or external validation are more defensible than complex hybrids supported only by random-split benchmark gains [11,24,26,31,33]. The full architecture–attention–fusion synthesis, including common failure modes and practical implications, is retained in Supplementary Table S7.

## 4. Discussion and Clinical Interpretation

This section discusses representative evidence on model architectures, attention, multimodal fusion, validation, reproducibility, computational requirements, and clinical relevance. The discussion does not rank models across studies because differences in datasets, tasks, validation designs, and metrics prevent direct comparison; instead, it evaluates the strength and clinical relevance of the reported evidence. Table 8, Table 9 and Table 10 summarize selected architectures, attention mechanisms, and fusion strategies, respectively, while Table 11 summarizes key validation and reporting safeguards. These tables are illustrative rather than exhaustive. The complete 60-study inventory is provided in Supplementary Table S2, with additional detailed evidence in Supplementary Tables S8–S10.

### 4.1. Architectural Choices

Architecture families should be interpreted as task-dependent choices rather than a ranking. Molecular GNNs provide transparent chemical baselines, dual-view models add pair context, knowledge graphs add mechanistic entities, and transformers or hypergraphs capture long-range or higher-order relations [11,13,18,23,26]. Added complexity is credible only when graph provenance, simpler baselines, component ablations, leakage-aware validation, and resource use are reported.

### 4.2. Attention Interpretation

Attention mechanisms help a model identify which parts of the drug information are more important for making a prediction. However, attention should be considered a way to understand model decisions, not proof that a specific biological mechanism causes a drug–drug interaction. Attention may highlight important molecular substructures, relationships between drugs, biological pathways, or different types of information used by the model. These highlighted features may sometimes reflect simple associations, repeated patterns in the data, or limitations of the training process rather than true pharmacological relationships [11,16,24,31].

Some attention methods consider the specific combination of two drugs, which is biologically reasonable because the factors influencing an interaction may depend on both drugs together rather than on each drug separately. However, these approaches can require more computational resources and may be affected by how drug structures or relationships are represented.

The biological reliability of these explanations has been studied less extensively than model prediction accuracy. Only a limited number of studies have compared attention-based explanations with known drug targets, enzymes, biological pathways, adverse-event information, or expert pharmacological knowledge [17,24,37,77,91]. Therefore, attention maps, highlighted drug features, and knowledge-graph connections should be considered supporting evidence or potential biological hypotheses rather than confirmed mechanisms. Future studies should validate explanations using biological evidence, feature-removal experiments, expert evaluation, and transparent reporting of both successful and unsuccessful explanations (Table 11).

### 4.3. Multimodal Fusion

Multimodal fusion is justified only when sources contribute distinct mechanisms: structure supplies chemistry, biological resources add targets and pathways, networks add relational context, and text may add unstructured evidence [17,31,32,33]. Concatenation should remain the baseline, and stronger claims about fusion should be supported by modality-ablation experiments, missing-modality tests, provenance checks, and external validation because additional modalities can introduce redundancy, missingness, and leakage [33,36]. The external validity of multimodal fusion models is discussed separately in Section 4.6 because most studies evaluated fusion strategies within individual benchmark datasets rather than across independent data sources.

### 4.4. Calibration and Reproducibility

Evaluation deserves particular attention because many DDI models are validated under conditions that do not match real medication-safety use. Random pairwise splits can place the same drug in training and test sets, allowing the model to learn drug identity, neighborhood popularity, or dataset-specific co-occurrence patterns rather than transferable interaction mechanisms. Drug-disjoint, temporal, and external validation settings are therefore essential when authors claim cold-start or prospective utility [8,9,22]. This point affects how readers should interpret nearly every performance comparison in the field.

#### 4.4.1. Uncertainty Calibration

Uncertainty calibration was much less common than discrimination reporting. Only 4 of 60 studies (6.7%) mentioned uncertainty or calibration. Probability-calibration methods and confidence scores were each reported in two studies (3.3%). Probabilistic approaches were reported in three studies (5.0%), while ensemble-based uncertainty was reported in one study (1.7%). Categories were non-exclusive, and the complete audit is provided in Supplementary Table S5. High AUROC or accuracy does not ensure reliable probabilities, so discrimination and calibration should be reported separately [28,92].

Representative approaches included temperature scaling, ensembles, and variational inference [27,59,72]. Future studies should report calibration curves or error measures and uncertainty intervals, and should evaluate whether calibrated thresholds support prioritization without increasing alert fatigue.

#### 4.4.2. Reproducibility

Code was available for 31 of 60 studies (51.7%), but complete preprocessing scripts, fixed partitions, configurations, software versions, and checkpoints were uncommon. Although all studies named an implementation framework and 59 reported some hyperparameters, only 38 (63.3%) reported all key settings; GPU hardware was specified in 8 (13.3%). Supplementary Table S5 provides the full audit.

Reproducibility also depends on identifier mapping, duplicate removal, graph construction, and negative sampling, not code availability alone [8,9,22,93]. Versioned data, executable preprocessing, fixed splits, seeds, and checkpoints should therefore accompany model code; minimum requirements are summarized in Table 11.

### 4.5. Computational Cost and Deployment Implications

The reporting audit indicates that deployment claims cannot be judged from predictive performance alone. Lightweight GAT, KGAT, and simple-fusion baselines generally offer easier inference and auditing, whereas graph transformers, hypergraphs, masked autoencoders, architecture-search methods, 2D/3D encoders, and LLM-enhanced pipelines introduce additional computational, preprocessing, memory, latency, and model-maintenance costs [17,18,21,81,84]. Major bottlenecks include pairwise attention, large contrastive batches, conformer generation, knowledge-graph construction, and text-retrieval or embedding pipelines.

Table 12 summarizes these architecture-level trade-offs. Future studies should report hardware, preprocessing time, total and per-epoch training time, per-pair inference time, peak memory, parameter count, and model-version dependencies, together with efficiency–accuracy comparisons such as those reported for KA-DDI and DMFDDI [20,31].

**Figure 4:**
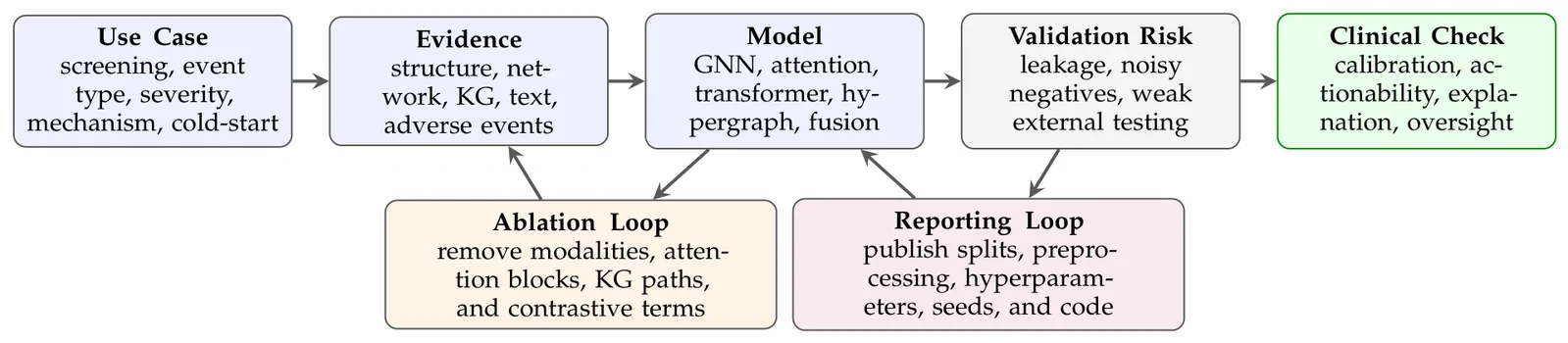
Decision map for selecting and validating a GNN-based DDI prediction pipeline. Model selection is linked to evidence availability, leakage-aware validation, ablation, and clinical actionability rather than benchmark accuracy alone.

The preceding findings are operationalized in Figure 4, which links use case, evidence, model choice, validation risk, ablation, reporting, and clinical checks. Table 13 maps these requirements to concrete DDI use cases, and Table 14 translates them into modality-specific preprocessing and validation checks.

### 4.6. External Validation and Generalizability of Multimodal Models

Multimodal models were usually tested within the same benchmark rather than across database versions or external resources, even though drug coverage, label definitions, and noise differ substantially between sources such as DrugBank and TWOSIDES [9,95]. The Multifaceted study illustrates the value of matched modality comparisons: its trimodal configuration outperformed fusion of all five modalities, reinforcing that complementarity and ablation matter more than modality count [33].

### 4.7. Interpreting Study-Level Performance Evidence

Reported performance values should be interpreted cautiously because the reviewed studies used heterogeneous datasets, prediction tasks, negative-sampling methods, class distributions, and validation protocols. These studies drew on resources such as DrugBank, TWOSIDES, KEGG, biomedical knowledge graphs, molecular-graph benchmarks, and text-derived DDI corpora. Although accuracy, F1 score, AUROC, AUPR, and ranking metrics were frequently reported, their numerical values are not directly comparable across differences in dataset construction, class balance, task definition, negative sampling, and validation design. Supplementary Table S2 provides the verified study-level evidence inventory and reported evaluation metrics. Accordingly, these study-specific results should not be interpreted as a direct cross-study ranking of model performance [6,8,9,22].

### 4.8. Practical Interpretation Scenarios for DDI Predictions

Predictions should be interpreted according to mechanism. For chemically driven DDIs, highlighted fragments can support follow-up review but should be linked to enzymes, transporters, targets, pathways, or adverse-event evidence before being considered actionable [11,24]. For pharmacokinetic or pharmacodynamic interactions, heterogeneous KGs and text-enhanced models may provide more relevant mechanistic context than molecular similarity alone [13,21,37].

For cold-start or rare interactions, confidence should remain low unless the model was evaluated with drug-disjoint, temporal, or external data [20,25,26]. Case reports should state whether the pair was absent from training and whether the explanation relies on transferable chemistry, biomedical context, or both.

Clinical outputs should be concise and actionable: predicted interaction type and severity, major supporting evidence, uncertainty, external-validation status, and a recommended next step [8,22,28]. This evidence trail is more useful than a dense attention visualization and motivates the recommendations that follow.

**Summary from the review evidence:** For RQ1, graph construction, modality selection, and validation design strongly shape reported performance. For RQ2, attention mechanisms and multimodal fusion can add value, but convincing evidence requires ablation, transparent preprocessing, and leakage-aware evaluation. For RQ3, high benchmark scores alone are insufficient for clinical readiness. The conclusions are therefore methodological rather than clinical: GNN-based DDI prediction is promising for prioritization and hypothesis generation, but deployment-oriented claims require standardized benchmarks, external or temporal validation, calibrated risk estimates, validated explanations, and transparent, reproducible pipelines.

Regarding RQ2, the synthesis of the included studies identified seven overlapping attention categories and ten non-exclusive multimodal-fusion or integration categories (Table 5 and Table 6). Because these categories overlap and the studies differed substantially in datasets, tasks, validation splits, and reported metrics, a pooled estimate of the performance effects of attention or fusion was neither appropriate nor defensible [34,35,80]. Within-study comparisons nevertheless provided conditional evidence of added value. SA-DDI evaluated its substructure-interaction component through a same-split ablation; DMFDDI compared attention-weighted fusion with a concatenation baseline; DSIL-DDI evaluated contrastive attention under an unseen-drug split; and the Multifaceted study found that a trimodal combination of text, image, and molecular-formula evidence outperformed fusion of all five evaluated modalities within its benchmark [25,30,31,33].

Collectively, these findings indicate that attention and multimodal fusion should be regarded as demonstrably useful only when improvements over matched simpler baselines are supported by component- or modality-level ablation and retained under leakage-aware, drug-disjoint, or external evaluation. Additional model complexity is not warranted when the contributing modalities are redundant, noisy, incomplete, or potential sources of information leakage.

### 4.9. Datasets, Biomedical Resources, and Validation Gaps

DDI prediction studies predominantly rely on a recurring set of benchmark resources, particularly DrugBank, TWOSIDES, KEGG, DDInter, molecular-structure collections, and biomedical knowledge graphs. Because individual studies often combine several resources and use different database versions, simple dataset-frequency counts can obscure overlap and are not treated here as mutually exclusive study categories. The increasing use of resources such as DRKG, Hetionet, and PrimeKG reflects a broader trend toward incorporating biological context beyond molecular structure alone.

These biomedical resources are complementary rather than interchangeable. DrugBank and DDInter primarily provide curated drug-pair interactions, descriptions, severity information, or reported mechanisms; TWOSIDES contributes pharmacovigilance-derived adverse-event signals but is more susceptible to reporting bias and confounding; and KEGG supplies pathway and molecular-network context. Heterogeneous knowledge graphs extend these sources by connecting drugs with proteins, genes, enzymes, diseases, pathways, phenotypes, and adverse events, as illustrated by resources such as PrimeKG [5,6,8,96]. The selected resource therefore determines the prediction target, graph topology, label provenance, missing-data pattern, and potential leakage pathways. However, the reviewed studies mostly fail to report database versions, identifier mapping, overlap among sources, label definitions, and whether external knowledge was constructed using information unavailable at the intended prediction date [9,22]. We discuss several important concerns that have not been sufficiently discussed in the current state-of-the-art.

#### 4.9.1. Incomplete and Noisy Labels

A pervasive challenge across all reviewed studies is the incompleteness and potential noise in DDI labels. Curated resources such as DrugBank, while considered gold standards, are inherently incomplete because they primarily capture well-studied interactions from FDA labels and peer-reviewed literature [5,6]. The absence of a recorded interaction does not necessarily indicate safety; it may reflect lack of testing, underreporting, or the recency of drug approval [9,22]. This creates a fundamental label-noise problem where models trained on these datasets may learn to distinguish “known” from “unknown” pairs rather than “interacting” from “non-interacting” pairs.

Incomplete labels can systematically bias model evaluation when unobserved drug pairs are treated as confirmed negatives rather than uncertain examples [9,22]. In the DDI context, this issue is exacerbated by the combinatorial growth in possible drug pairs as new drugs and combination therapies enter practice, which outpaces experimental and clinical testing capacity [4,5]. TWOSIDES, derived from FDA Adverse Event Reporting System data, is also susceptible to reporting bias and lacks a reliable denominator for total drug-pair exposure.

This concern is consistent with the broader limitations of computational interaction data summarized by Zhou et al. [97]: incomplete biological knowledge, heterogeneous evidence, and uncertain negative examples can constrain what an interaction-prediction model can learn. In DDI research, the implication is that an unrecorded drug pair should not automatically be interpreted as a verified non-interaction.

The risk-of-bias framework treats negative sampling as a distinct assessment domain (Table 2), but sensitivity analyses comparing alternative negative definitions were uncommon and inconsistently reported. This gap is concerning because random, similarity-based, or pharmacologically informed sampling can substantially alter model rankings and reported accuracy [9,22]. Future work should treat unobserved interactions as uncertain where appropriate and report sensitivity analyses across multiple sampling strategies.

#### 4.9.2. Data Leakage from Random Pairwise Splits

A second critical concern is leakage-prone evaluation design. Random pairwise splits were the dominant validation approach in the reviewed evidence, assigning drug pairs to training and test sets without requiring drug-disjoint partitions. Although the pair records differ, the same drug identities and highly overlapping neighborhoods can occur in both partitions, allowing models to exploit drug-specific interaction patterns rather than transferable mechanisms [9,22]. Message-passing architectures are especially vulnerable when shared drug nodes or neighborhoods allow information associated with training pairs to influence representations evaluated on nominally held-out pairs [9,22].

Only 3 of the 60 primary studies (5.0%) explicitly mentioned or addressed data leakage [21,31,40], whereas 57 (95.0%) did not discuss it. These figures measure reporting rather than confirmed leakage; paper IDs map to Supplementary Table S2, and the aggregate audit is provided in Supplementary Table S5.

Architectural innovations do not eliminate evaluation bias. For example, Flow2GNN enhances message passing for graphs beyond the homophily assumption [98]; however, even a sophisticated message-passing mechanism may exploit repeated drug identities or overlapping neighborhoods when random pairwise splits are employed. Leakage control must therefore be implemented through drug-disjoint, temporal, or external partitioning rather than inferred solely from architectural novelty.

Such designs may inflate benchmark performance and do not represent new-drug use, where models must rely on transferable chemical and biological evidence rather than memorized interaction patterns. Random-pair and drug-disjoint results should not be compared directly because task difficulty differs. Only a minority of studies reported drug-disjoint or cold-start validation, and few provided matched transductive and inductive results [24,25,26,37,77]; therefore, no pooled magnitude of this validation gap was estimated.

#### 4.9.3. Underexplored Cold-Start Evaluation

Cold-start evaluation, where models must predict interactions for drugs not seen during training, is critical for clinical translation because new drugs enter practice continuously. Systematic cold-start experiments remain underrepresented, and the reported configurations vary substantially: some test one unseen and one known drug, others test two unseen drugs, and still others use structure- or scaffold-based separation [20,25,26,37,51,54,68,71,74,76]. Results from these settings should therefore be reported separately rather than combined into a single cold-start estimate [22,26].

The scarcity of cold-start evaluation is particularly concerning because results can change substantially when models move from transductive testing on previously observed drugs to inductive testing involving unseen drugs. Direct comparisons in DSN-DDI and DSIL-DDI demonstrate the importance of reporting these settings separately, while broader review evidence identifies cold-start generalization as a persistent challenge in DDI prediction [22,25,26]. Because the studies use different datasets, unseen-drug definitions, tasks, and metrics, a pooled percentage decrease is not calculated here. Nevertheless, the reduced and more variable performance under unseen-drug evaluation indicates that strong random-split results may partly depend on previously observed drug identities and interaction neighborhoods rather than fully transferable chemical and biological mechanisms.

Weighted local-information augmented GNNs, as demonstrated by Meng et al. [99] for drug repositioning, offer a potentially useful direction for cold-start generalization. Their approach, which emphasizes augmented local graph information, could be adapted to DDI prediction. Promising cold-start designs use contrastive objectives [20,25,27], substructure-aware attention [11,26,30], and multi-view representations that reduce dependence on drug identity [48,71]. Because unseen-drug settings and metrics differ across studies, no pooled cold-start accuracy is reported here.

#### 4.9.4. Temporal Validation Gaps

Temporal validation occurs less frequently than cold-start evaluation. Only a limited number of studies have reported future-oriented analyses in which historical evidence is separated from subsequent interaction evidence [77]. DDI-GPT also offers a related analysis using future data; however, it was included solely as contextual preprint evidence [88]. This scarcity presents a significant challenge because the interaction landscape evolves continuously as new evidence emerges, interaction classifications are updated, and new drugs receive approval [9].

A central methodological challenge in temporal validation is accurate timestamping, which requires version-controlled databases and explicit cutoff dates [9]. For DDI prediction, temporal splits require (1) database versioning to establish discovery dates, (2) exclusion of future evidence from feature construction (e.g., not using knowledge graphs containing interactions discovered after the cutoff), and (3) careful handling of interactions that were known but not yet curated into the reference database.

Network-embedding studies in adjacent biomedical tasks, such as PrGeFNE for disease-gene prediction [100], demonstrate the value of learning from heterogeneous biomedical networks; however, they do not substitute for future-facing DDI evaluation. A temporal DDI experiment must preserve chronology throughout labels, features, knowledge-graph construction, and literature-derived inputs to prevent future information from leaking into the training representation.

The lack of temporal validation reduces the practical value of graph neural network (GNN)-based methods, especially for drug–drug interaction (DDI) prediction in real-world pharmacovigilance. Clinical decision support systems must remain robust as new evidence becomes available. Without temporal evaluation, it is unclear whether strong benchmark results reflect genuine predictive ability or overfitting to known interactions. We recommend that future studies include temporal splits in addition to drug-disjoint splits, and at minimum, report performance on interactions identified in the most recent one to two years of database releases. Table 15 summarizes the principal evaluation protocols and reporting requirements.

Benchmark fragmentation compounds these validation gaps. Stereochemistry-aware systems such as LSA-DDI introduce specialized 3D feature fusion and contrastive cross-attention [101], but study-specific datasets, database versions, split definitions, and metrics do not provide a standardized basis for comparison with graph-only, multimodal, or LLM-enhanced methods. Shared benchmark versions, fixed leakage-aware partitions, and common calibration and efficiency measures are therefore needed alongside architecture-specific evaluations.

#### 4.9.5. LLM Integration Gaps

LLM-enhanced fusion represents an emerging but heterogeneous direction. Included and contextual studies use language or foundation models for semantic embeddings, synthetic text augmentation, structured extraction, or KG paths converted to text [21,52,88,89]. DDI-GPT integrates biomedical knowledge graphs with pre-trained LLMs to predict DDIs, supports zero-shot evaluation on newly curated FDA interaction data, and provides gene and network-level evidence to aid interpretation [88]. However, its incremental benefit over matched graph-only or simpler language-model baselines requires further evaluation under consistent datasets, validation protocols, computational budgets, and leakage controls.

More broadly, evidence for the incremental value of LLM components remains limited. Few studies compared LLM-derived features with simpler text encoders or graph-only baselines while holding datasets, validation splits, optimization procedures, and computational budgets constant. Consequently, reported improvements cannot always be attributed specifically to the LLM component. LLM integration also introduces additional sources of methodological variation, including prompt design, model and version changes, retrieval-corpus selection, decoding settings, access date, and potential training-data leakage. Rigorous evaluation therefore requires controlled ablations, complete reporting, reproducible workflows, and explicit leakage safeguards [21,88,90]. This evidence gap is consistent with broader methodological concerns in biomedical LLM research. The TRIPOD-LLM guideline emphasizes transparent reporting, reproducibility, human oversight, and task-specific performance evaluation for biomedical studies using LLMs [90]. For DDI prediction, we identify four critical gaps in LLM integration:

1. **Missing modality ablations**: LLM-free comparisons were uncommon, making it difficult to determine whether language-model features provide genuine added value or merely correlate with existing inputs [21,89].
2. **Version and prompt instability**: LLM behavior can change with model version, prompt phrasing, temperature settings, and access date, yet systematic sensitivity analyses were generally absent.
3. **Leakage risks**: LLMs pre-trained on large biomedical corpora may have encountered benchmark-related evidence during training; explicit leakage testing was generally not reported.
4. **Computational trade-offs**: LLM-enhanced approaches introduce memory, inference-time, and possible API costs, while efficiency–accuracy trade-offs were rarely quantified.

## 5. Recommendations for Model Design Selection

Building on the synthesis above, this section translates the findings from 60 primary graph-based or graph-enhanced studies, together with contextual evidence from broader DDI-methodology reviews, into practical recommendations for selecting fusion strategies, attention mechanisms, and GNN architectures according to use case, data availability, validation requirements, and deployment constraints [6,8,22]. Reported numeric improvements are cited only as original-study findings under their specific datasets, split designs, and comparator baselines; stand-alone endpoint scores are not treated as transferable evidence or as pooled estimates of model superiority.

### 5.1. Recommendation 1: Match Architecture to Task Complexity

For **binary classification** with homogeneous features, simple concatenation with a basic GAT provides a reasonable baseline. In its original evaluation, MDG-DDI reported an F1 improvement in an inductive setting using concatenated structural and semantic features; that gain should be read only against the dataset, split, and comparator baselines used in the primary study [63]. For **multi-class** (65–86 types) or **multi-label** (200–963 types) prediction, attention-weighted or bi-level fusion should be considered, but only after comparing against concatenation and single-modality baselines. DMFDDI’s reported F1 improvement over concatenation is useful within its original multi-type benchmark, but it should not be transferred to other datasets or split designs without re-evaluation [31]. MUFFIN reported strong results across three tasks using bi-level fusion, but these findings should still be interpreted within the datasets and splits used by the primary study [87].

### 5.2. Recommendation 2: Select Attention Based on Interaction Type

- **Molecular graph encoding:** Use GAT when atom, bond, and fragment patterns are the primary evidence. SSI-DDI supports this choice through a molecular-substructure benchmark, but its reported AUPRC should be interpreted only with the original dataset, split, and baseline set [11].
- **Drug-pair interaction:** Use co-attention when pair-specific substructure interactions are central. SA-DDI’s substructure-interaction ablation is informative because it compares the same dataset and split with and without the SSIM component, rather than relying on an isolated accuracy value [30].
- **Heterogeneous entity integration:** Use hierarchical attention (node + meta-path) when drugs are connected to multiple biomedical entity types. HAN-DDI should be compared only against baselines evaluated on the same heterogeneous graph, meta-path design, and data split [83].
- **Long-range dependencies:** Use transformer self-attention when global molecular or multimodal dependencies are expected. SGT-DDI’s reported performance is meaningful only alongside the original 2D/3D dataset, split, and 2D-only or 3D-only comparator baselines [17].
- **Modality importance:** Use attention feature weighting when the goal is to test whether modalities contribute unequally. DMFDDI is most useful as within-study evidence because it compares against a concatenation baseline under the same benchmark protocol [31].
- **Low-data / cold-start:** Use contrastive attention when unseen-drug generalization is the target. DSIL-DDI’s improvement should be read in the context of its unseen-drug split and the baselines reported in the original study [25].

### 5.3. Recommendation 3: Choose GNN Architecture by Data Availability

- **Basic structure-based:** GAT/GCN models are often sufficient when molecular-structure or DDI-network evidence is limited. MIRACLE illustrates this setting, but its low-label findings should be checked against the original training fraction, split, and baseline protocol [12].
- **Inductive/cold-start:** Dual-view architectures are more appropriate when new-drug or new-new-drug splits are the intended deployment setting. DSN-DDI’s accuracy should therefore be interpreted as cold-start evidence tied to its original split and baselines, not as a general endpoint score [26].
- **Rich biomedical KGs:** Heterogeneous GNNs are suitable when reliable KG entities and relations are available. KnowDDI’s sparse-KG experiments are useful because they test sensitivity to missing graph evidence within the same benchmark context [24].
- **2D+3D or multiscale fusion:** Graph transformers are relevant when conformational or multiscale molecular evidence is justified. ALG-DDI’s future-DDI analysis should be treated as a small external-style case study, not as a replacement for broad temporal validation [77].
- **Higher-order relationships:** Hypergraph architectures are appropriate when interactions depend on higher-order drug–entity relations. MFHNN-DDI’s reported accuracy should be read with its hypergraph construction rule, dataset, split, and comparator baselines [18].
- **Unknown optimal architecture:** Use automated graph neural architecture search (GNAS) when the optimal architecture is unknown. AutoDDI discovers dataset-optimal depths (5–8 layers) [81].

### 5.4. Recommendation 4: Prioritize Contrastive Learning for Generalization

When labeled data are limited or **out-of-distribution generalization** is required, contrastive learning is often useful, provided that positive pairs, negative pairs, and augmentations reflect valid biomedical invariances. In original-study evaluations, DSIL-DDI and MRGCDDI reported improvements over their stated baselines under their respective unseen-drug and SimGRACE benchmark settings [25,68]. CASE maintained robust performance under its reported imbalance setting [27], and KA-DDI reported robustness across sparse (DrugBank) and dense (KEGG) networks [20]. These findings should be interpreted as dataset-, split-, and baseline-specific evidence for contrastive objectives rather than as pooled effect sizes.

### 5.5. Recommendation 5: Leverage LLMs for Text-Rich Applications

When **unstructured text data** (biomedical literature, clinical reports) are available, LLM-enhanced fusion can be useful as an evidence-extraction or representation layer, but it should not replace structured graph validation. In original-study evaluations, DDI-GPT reported an AUROC gain relative to its comparator in a zero-shot setting on new FDA data [88], BioFocal-DDI reported improved minority-class F1 after BioGPT-based augmentation [52], and a multi-feature extraction pipeline reported high structured-extraction accuracy using GPT-4o [89]. As identified in Section 4.9.5, LLM behavior can change with model version, prompt design, retrieval corpus, access date, dataset construction, and leakage controls; therefore, these findings require exact prompts, model versions, extraction rules, benchmark splits, and comparator baselines before they can be interpreted. Accordingly, we recommend the following practices for future LLM-enhanced DDI prediction studies:

- Report exact model name, version, and access date for all LLMs;
- Provide full prompts, temperature settings, and decoding parameters;
- Include modality ablations comparing LLM-enhanced to LLM-free predictions;
- Conduct leakage checks by testing on interactions discovered after the LLM’s training cutoff;
- Report computational costs including token counts, API costs, and inference latency;
- Validate extraction quality with manual review or comparison to structured databases.

### 5.6. Recommendation 6: Match Architecture to Deployment Constraints

- **Resource-constrained (IoMT, edge):** Use lightweight architectures. DDI-KGAT uses only 32-dimensional embeddings [84]. KITE-DDI requires ∼4 GB of memory [56].
- **Accuracy-prioritized benchmark research:** Consider hybrid approaches combining multiple attention types, but require ablation and cost reporting. HTCL-DDI, KGMAEDDI, and SGT-DDI are representative high-performing examples [17,32,71].
- **Clinical adoption:** Prioritize calibrated predictions and auditable evidence trails. KnowDDI’s explanatory paths and SGT-DDI’s attention maps provide mechanistic hypotheses, but they require pharmacological validation before clinical use [17,24].

### 5.7. Recommendation 7: Avoid Over-Fusion and Validate via Ablation

When modalities are **redundant**, over-fusion degrades performance. In the multifaceted study, the trimodal configuration (text, image, and formula) outperformed the five-modality configuration within its own benchmark protocol [33]. MOPDDI’s orthogonal-projection results also show that removing features useful for new drugs can harm inductive performance, but the magnitude of that loss depends on the original Task 3 dataset, split, and baseline design [36]. AutoDDI’s key insight that optimal architecture varies by dataset underscores the importance of ablation studies [81].

Table 16 integrates the preceding design recommendations into a concise decision framework for matching evidence sources, model families, and validation requirements.

The optimal model design depends critically on task type, data availability, and deployment constraints [6,8,22]. The growing trend toward **hybrid approaches** combining complementary fusion strategies, attention mechanisms, and GNN architectures represents a prominent 2025–2026 research direction [17,32,77]. However, hybrid complexity should be justified through ablation, cost reporting, calibration, and external validation rather than assumed to be preferable.

### 5.8. A Practical Guide for Clinicians: How to Access and Use GNN-Based DDI Prediction Tools

Clinicians should not currently be expected to build or independently run GNN models for routine DDI screening. Most GNN-based systems in the reviewed literature remain research prototypes requiring programming expertise, model-specific data preparation, source code, software dependencies, and pre-trained model parameters. Their predictions have generally been evaluated on retrospective benchmark datasets and should not be used independently to initiate, discontinue, or modify treatment [9,22,28].

#### 5.8.1. Established Interaction Resources

Routine screening should begin with an interaction checker integrated into the electronic prescribing or pharmacy system, or with an authoritative drug-information resource. The access and licensing information below was verified on 30 July 2026; however, availability and access conditions may change.

- **DDInter 2.0** is an open-access database available without login at https://ddinter2.scbdd.com/ (accessed on 30 July 2026). It provides interaction descriptions, risk levels, mechanisms, and management information [102].
- **The University of Liverpool interaction checkers** provide freely accessible, evidence-based interaction information for HIV, PrEP, hepatitis, and COVID-19 at https://www.druginteractions.org/ (accessed on 30 July 2026) [103].
- **Medscape** provides an online checker for medicines and supplements at https://reference.medscape.com/drug-interactionchecker (accessed on 30 July 2026) [104].
- **DrugBank Clinical, UpToDate Lexidrug (formerly Lexicomp), and Micromedex** provide curated drug-information or interaction services, but an individual account, institutional subscription, or commercial license may be required [105,106,107].

These resources differ in coverage, evidence grading, update schedules, and intended use. Clinicians should therefore follow institutional policy and confirm whether a resource is authorized for the relevant clinical setting.

#### 5.8.2. A Simple Clinical Workflow

1. Enter the patient’s complete medication list, including prescription medicines, over-the-counter products, and supplements, into a trusted interaction checker.
2. Review the reported severity, mechanism, evidence, dose, timing, treatment duration, and monitoring recommendations.
3. Assess patient-specific factors, including age, renal or hepatic function, comorbidities, and the feasibility of clinical or laboratory monitoring.
4. Confirm clinically important findings through a pharmacist or clinical pharmacologist, the product label, an institutional guideline, or a second authoritative source before changing treatment.

Research tools serve a different purpose. Published examples include DDI-GCN, an explainable graph-convolutional model, and DDI-Pred, a freely accessible structure-based prediction service at https://way2drug.com/ddi/ (accessed on 30 July 2026) that explicitly states that it is not a clinical interaction checker [108,109]. Research-server availability may change, and access to a website or source code does not establish clinical validity.

When a GNN model suggests a previously unreported interaction, the result should be treated as a hypothesis requiring pharmacological and clinical verification rather than as a definitive recommendation. Current GNN systems are therefore more appropriate for research prioritization, pharmacovigilance triage, and decision-support development than for independent bedside use. Table 17 summarizes the practical distinctions among these resource types.

## 6. Future Research Directions

Future research should move beyond isolated benchmark optimization toward a broader translational pipeline for DDI prediction. At the data and evaluation level, the field needs standardized and openly documented benchmark suites, harmonized task definitions, transparent negative-sampling protocols, temporal splits that reflect drug-approval timelines, cross-database validation, and explicit cold-start testing for unseen drugs, rare interactions, and underrepresented drug classes [6,8,22,34,35]. Shared preprocessing code, fixed data partitions, reporting checklists, and reproducible baselines would make performance comparisons more reliable and reduce the risk that reported improvements arise from leakage, duplicated evidence, or dataset-specific shortcuts.

At the modeling level, future GNN-based systems should integrate molecular graphs, DDI networks, biomedical knowledge graphs, omics evidence, pharmacokinetic and pharmacodynamic attributes, adverse-event profiles, and biomedical text in a principled manner rather than simply adding more modalities. Promising directions include uncertainty-aware graph transformers, calibrated hypergraph models, contrastive and self-supervised pre-training for low-data regimes, foundation-model-assisted feature extraction, and architecture-search methods that explicitly balance accuracy, efficiency, robustness, and interpretability [17,20,32,77,81]. Because multimodal and LLM-enhanced models may absorb unsupported textual associations or hidden benchmark labels, future studies should report modality ablations, missing-modality stress tests, provenance of external knowledge, and safeguards against information leakage [17,21,33,88].

At the clinical and regulatory level, DDI prediction should be evaluated as a decision-support problem rather than only as a pair-classification problem. Models should produce calibrated risk scores, uncertainty estimates, mechanistic explanations, and clinically meaningful evidence trails that connect predicted interactions to targets, enzymes, pathways, transporter mechanisms, pharmacokinetic interactions, or adverse-event phenotypes [24,28,37]. Prospective validation, external hospital or pharmacovigilance datasets, human expert review, fairness assessment across populations and medication classes, and lightweight deployment studies will be essential before these systems can support medication-safety workflows. Fairness assessment is especially important because DDI labels derived from the literature, product labels, spontaneous reports, or hospital records may underrepresent pediatric patients, pregnancy and lactation, older adults with extreme polypharmacy, people with multimorbidity, and populations from low-resource health systems. Future benchmarks should therefore report drug-class, severity, demographic-context, and data-source coverage where such metadata are available. In this broader direction, GNN-based DDI prediction can evolve from benchmark-centered model development toward trustworthy, explainable, and clinically actionable pharmacological risk assessment. Table 18 outlines a staged pathway for connecting model development to realistic medication-safety use.

## 7. Limitations of This Review

This review has several limitations. First, the search was restricted to IEEE Xplore, ScienceDirect, PubMed, and Google Scholar, and only the first 100 relevance-ranked Google Scholar results were examined. Studies indexed only in other databases or search platforms, or described using different terminology, may therefore have been missed. Second, the review was not prospectively registered, and no separate protocol was published, although the final eligibility criteria, extraction items, and PRISMA process are reported in the manuscript. Third, graph-based prediction, event classification, and graph-supported extraction tasks were synthesized together because they share common representation and validation issues. Although this broadened the methodological coverage, it limited direct comparisons across task types.

Fourth, the included studies varied substantially in dataset construction, negative sampling, validation design, reported metrics, and outcome definitions. Quantitative pooling was therefore not appropriate, and the study-specific results reported in Supplementary Table S2 should not be interpreted as a model ranking [6,8,22]. Fifth, potential publication and reporting biases were considered qualitatively rather than assessed statistically because no pooled effect estimate was calculated. The available evidence may therefore favor strong positive findings, while failed external validations, negative results, and unfavorable ablation findings may be underreported [35,80]. Sixth, this review focused on graph-based and graph-enhanced neural methods; traditional machine-learning approaches, rule-based pharmacological resources, and non-graph deep-learning methods were discussed only as contextual evidence rather than reviewed comprehensively [7,9,28].

Beyond these review-level limitations, the underlying evidence base remains constrained by incomplete or noisy labels, leakage-prone data splits, limited unseen-drug and temporal evaluation, sparse reporting of calibration and computational requirements, weak reproducibility, and insufficient validation of attention-based, multimodal, and LLM-derived explanations [6,8,9,22,28,90]. These literature-level concerns, discussed in detail above, highlight the need for standardized and versioned benchmarks, drug-disjoint and temporal validation, calibrated uncertainty estimates, reproducible pipelines, biological or expert validation of explanations, component-level ablation studies, and prospective or independent external evaluation [9,22,95].

## 8. Conclusions

This systematic review shows that graph-based neural models have become a central approach for computational drug–drug interaction prediction, combining molecular structures, interaction networks, biomedical knowledge graphs, attention mechanisms, and multimodal fusion. However, only a small minority of studies explicitly discussed data leakage, and random pairwise partitions can allow the same drug identities to recur across training and test sets; comparatively few studies rigorously evaluated entirely unseen drugs or interactions discovered after a defined temporal cutoff. Attention mechanisms and multimodal fusion are promising for identifying relevant molecular features and combining complementary evidence, but they require ablation studies and pharmacological validation rather than being treated as definitive explanations. For clinical use, models must provide calibrated risk estimates, validated explanations, reproducible preprocessing pipelines, and evidence from prospective clinical workflow evaluation or independent external validation. GNN-based DDI prediction is therefore valuable for hypothesis generation and pharmacovigilance triage, but it is not yet ready for independent clinical decision-making.

## Figures and Tables

**Table 1:** Search strings and database record counts used to construct the PRISMA flow diagram.

Source	Search String	Filters	Records Identified
IEEE Xplore	(“drug–drug interaction” OR DDI) AND (“graph neural network” OR GNN OR “knowledge graph” OR hypergraph OR “graph attention”)	Document type: peer-reviewed conference papers, journal articles, and early-access articles; publication years: 2021–2026; final search update: 15 March 2026	74
ScienceDirect	(“drug–drug interaction prediction” OR “drug interaction prediction”) AND (GNN OR “graph neural network” OR “knowledge graph” OR “multimodal fusion”)	Years: 2021–2026; article type: research article; subject areas: computer science, pharmacology, biochemistry, genetics and molecular biology; final search update: 15 March 2026	96
PubMed	(“drug–drug interaction” OR DDI) AND (“graph neural network” OR GNN OR “knowledge graph” OR “multimodal fusion”)	Publication years: 2021–2026; English-language records; preprints excluded; final search update: 15 March 2026	67
Google Scholar	“drug–drug interaction prediction” AND (“graph neural network” OR GNN OR “knowledge graph” OR graph)	Publication years: 2021–2026; first 100 relevance-ranked results examined; 53 retained after date, document-type, and scope filtering; final search update: 15 March 2026	53
**Total records identified in the PRISMA flow** [34,35]			**290**

**Table 2:** Risk-of-bias and applicability criteria used to structure the narrative synthesis [8,22,34,35].

Domain	Lower Concern	Higher Concern	Relevance to DDI Prediction
Train–test separation	Drug-disjoint, temporal, or external splits; duplicate evidence removed	Random pairwise split only; same drugs or duplicated evidence appear across splits	Random splits can inflate performance and hide cold-start failure.
Negative sampling	Transparent sampling; unknown interactions treated cautiously; sensitivity analysis reported	Unobserved pairs treated as true negatives without justification	DDI databases are incomplete, so false negatives can distort training and evaluation.
Generalization design	Cold-start, cross-dataset, temporal, or external validation included	Only internal benchmark validation reported	Clinical deployment requires performance on new drugs, new evidence, and future interactions.
Baselines and ablations	Strong baselines, modality ablations, and component-level tests reported	Only favorable comparisons or incomplete ablations reported	Ablations help determine whether gains come from graph modeling, attention, fusion, or data leakage.
Reproducibility	Code, data splits, hyperparameters, and preprocessing steps available	Limited implementation detail or unavailable splits/code	Reproducibility is required for fair comparison and clinical trust.
Interpretability	Explanations linked to substructures, targets, pathways, enzymes, or adverse events	Attention maps or case studies reported without pharmacological validation	Interpretability claims should support mechanistic plausibility rather than only visualization.
Clinical applicability	Reports calibration, uncertainty, severity, actionability, or workflow relevance	Focuses only on benchmark accuracy	Medication-safety use requires calibrated, actionable, and clinically meaningful predictions.

**Table 3:** Study-level risk-of-bias and applicability summary for the 60 included primary studies.

Overall Concern Group	Included Studies	Main Reason for Classification
Lower concern	DSIL-DDI [25]; DSN-DDI [26]; KA-DDI [20]; MOPDDI [36]; CASE [27]; Interpretable graph model [37]	Reported stronger evidence for generalization, ablation, external or future-facing validation, interpretability, robustness, or reproducible design relative to the rest of the evidence base.
Moderate concern	SSI-DDI [11]; Taco-DDI [16]; SGT-DDI [17]; MFHNN-DDI [18]; FG-DDI [19]; LLM-DDI [21]; DMFDDI [31]; KGMAEDDI [32]; Multifaceted fusion model [33]; SA-DDI [30]; SynCaps-DDI [38]; ImageDDI [39]; KnowDDI [24]; Multimodal KG-DNN [40]; MRLF-DDI [41]; MEDL-DDI [42]; KGDB-DDI [43]; RCAN-DDI [44]; AI-DDI [45]; SCATrans [46]; DrugDAGT [23]; GMIA [47]; DGNN-DDI [48]; DDI-MuG [49]; SRR-DDI [50]; IIB-DDI [51]; BioFocal-DDI [52]; Memory interaction NN [53]; EDDINet [54]; MMDDI-SSE [55]; KITE-DDI [56]; HDN-DDI [57]; Spatial-aware KG capsule model [58]; Contrastive hypergraph model [59]; HLN-DDI [60]; 3D elastic MPNN model [61]; HGNN-DDI [62]; MDG-DDI [63]; MGDDI [64]; Multi-view contrastive model [65]; MMFF-DDI [66]; Hierarchical multi-relational model [67]; MRGCDDI [68]; DualC [69]; MSHGCL [70]; HTCL-DDI [71]; MAVGAE [72]; Multi-view interpretable model [73]; MPHGCL-DDI [74]; SSF-DDI [75]; KG contrastive method [76]; ALG-DDI [77]	Reported useful model or benchmark evidence but commonly retained at least one concern, such as random pairwise validation, incomplete negative-sampling justification, limited external validation, incomplete reproducibility details, or interpretability claims not independently validated.
Higher concern	GCN collaborative-filtering model [78]; Multi-dimensional interaction network [79]	Provided relevant graph-based DDI-prediction evidence but retained greater concern because of limited information for leakage-aware, reproducible, or deployment-oriented assessment.

**Table 4:** Comparative synthesis of major model families for GNN-based DDI prediction [11,13,17,18,21].

Model Family	Main Strength	Common Limitation	Methodological Guidance
Molecular-graph GNNs	Capture atom, bond, and substructure information directly from chemical structure	May miss enzyme, transporter, pathway, and patient-context mechanisms	Best suited for chemically driven DDIs; should be paired with mechanism-aware validation.
DDI-network GNNs	Exploit interaction topology and higher-order drug neighborhoods	Sensitive to missing labels and topology leakage in random pairwise splits	Should report cold-start or drug-disjoint results, not only transductive benchmarks.
Knowledge-graph and heterogeneous GNNs	Connect drugs to proteins, genes, diseases, pathways, side effects, and phenotypes	Depend on KG completeness, relation quality, and meta-path choices	Useful for mechanistic plausibility, but relation provenance and ablations are essential.
Attention and graph-transformer models	Model pair-specific relevance, long-range dependencies, and cross-modal interactions	Attention weights can be overinterpreted as explanations	Attention should be validated with ablation, substructure checks, or pharmacological evidence.
Hypergraph and fragment-aware models	Represent higher-order relations among fragments, pathways, or multi-entity interactions	Hypergraph construction choices can be dataset-specific and difficult to compare	Strong when interactions are not purely pairwise, but construction rules must be transparent.
Contrastive/self-supervised models	Improve representation learning for sparse labels and cold-start settings	Performance depends on augmentation design and positive/negative pair assumptions	Most valuable when pre-training or view alignment reflects real biomedical invariances.
Multimodal and LLM-enhanced models	Combine chemical, biological, network, text, and clinical evidence	Risk of over-fusion, missing-modality bias, and hidden label leakage from external text	Require modality ablation, provenance reporting, and external validation before clinical claims.

**Table 5:** Principal attention-mechanism categories, defining characteristics, advantages, and limitations in DDI prediction [11,16,17,23,30,32]. Categories are non-exclusive.

Attention Category	Defining Characteristic	Advantages	Limitations
Graph Attention (GAT)	Learns neighbor-importance weights	Interpretable local focus on atoms, fragments, or graph neighbors	Drug-order sensitivity and cold-start degradation
Cross-/Co-Attention	Models cross-drug or cross-modal interactions	Identifies bidirectional pair and substructure interactions	Quadratic complexity and possible order sensitivity
Hierarchical Attention	Weights nodes, semantic levels, or meta-paths	Captures multiple semantic scales and supports meta-path interpretation	Manual meta-path design and overfitting risk
Knowledge-Graph Attention (KGAT)	Weights knowledge-graph neighbors and relations	Prioritizes multiple entity types and relation-aware evidence paths	Dependence on KG quality; cold-start limitations persist
Transformer Self-Attention	Applies global attention across nodes or modality tokens	Captures long-range dependencies and supports 2D/3D or cross-modal fusion	Large parameter counts and high data requirements
Feature/Modality Weighting	Learns feature-dimension or modality importance	Provides simple modality diagnostics and adaptive fusion	May miss fine-grained pairwise interactions
Contrastive Attention	Aligns complementary representation views	Improves view alignment and robustness under sparse or imbalanced data	Sensitive to negative sampling; often evaluated only on binary tasks

**Table 6:** Principal representation-integration and multimodal-fusion strategies, defining characteristics, advantages, and limitations in DDI prediction [17,31,32,33,36].

Fusion Category	Defining Characteristic	Advantages	Limitations
Simple Concatenation	Combines features without learned modality weighting	Simple, transparent, and reproducible baseline	Treats modalities equally and does not model cross-modal interactions
Attention-Weighted	Learns feature or modality importance	Provides dynamic weighting and interpretable importance scores	Global weights may suppress useful features or reflect noisy evidence
Cross-/Co-Attention	Models fine-grained cross-drug or cross-modal interactions	Aligns substructures, drug pairs, or complementary modalities	Quadratic complexity and possible order sensitivity
Bi-Level/Multi-Level	Integrates multiple representation granularities	Captures local and global evidence at several scales	High complexity and difficult component ablation
Masked Autoencoder	Uses reconstruction-driven representation alignment	Learns shared semantics from incomplete or masked inputs	Computationally intensive with a limited DDI evidence base
Hierarchical Transformer	Performs multiscale transformer-based fusion	Combines global context with hierarchical representations	Complex, parameter-intensive, and data-hungry
Orthogonal Projection	Separates shared and modality-specific information	Reduces redundancy between evidence sources	May remove signals useful for unseen or rare drugs
Graph-Based Integration	Uses a GNN to combine relational evidence; not necessarily multimodal	Preserves inherent biomedical structure and dependencies	Depends on graph quality and may face scalability limits
LLM-Enhanced	Combines text- or LLM-derived representations with structured evidence	Adds rich semantics and possible zero-shot transfer	Model-version dependence, black-box behavior, and provenance concerns
Contrastive	Aligns complementary representation views	Improves view consistency and robustness under imbalance	Sensitive to negative sampling; binary evaluations remain common

**Table 7:** Summary of computational cost and scalability reporting across the included studies.

Metric	Count	Percentage	Details
GPU Specified	8	13.3%	A100, V100, RTX 3090, RTX 2080 Ti, A40, RTX 2070 Ti
Training Time Reported	12	20.0%	Ranges from 35 s/epoch to 5 h
Inference Time Reported	6	10.0%	Ranges from 1 ms to 8–10 ms per sample
Parameter Count Reported	18	30.0%	Ranges from 4.1 M to 120 M
Memory Usage Reported	8	13.3%	Ranges from 4 GB to 9.5 GB
Scalability Analysis	15	25.0%	Limited training data, large-scale datasets, etc.

**Table 8:** Selected representative GNN architectures spanning molecular, dual-view, knowledge-graph, transformer, hypergraph, contrastive, and architecture-search approaches.

Family	Ref.	Model	Main Design	Methodological Relevance
Molecular GAT	[11]	SSI-DDI	Substructure GAT with co-attention	Representative chemically driven baseline.
Dual-view GNN	[26]	DSN-DDI	Intra- and inter-drug views	Representative unseen-drug design.
Pre-trained HGNN	[13]	HetDDI	Atom masking and KG link prediction	Adds heterogeneous biomedical context.
Knowledge subgraphs	[24]	KnowDDI	Adaptive KG edge refinement	Provides inspectable evidence paths.
Graph transformer	[23]	DrugDAGT	Local message passing plus global attention	Captures long-range molecular dependencies.
2D/3D transformer	[17]	SGT-DDI	Molecular graph and conformation fusion	Tests complementary geometric evidence.
Hypergraph model	[18]	MFHNN-DDI	Higher-order fragment relations	Represents interactions beyond pairwise edges.
Contrastive KG model	[20]	KA-DDI	Knowledge-adaptive contrastive sampling	Targets sparse and imbalanced settings.
Architecture search	[81]	AutoDDI	Graph neural architecture search	Demonstrates dataset-specific operator selection.

**Table 9:** Selected representative attention mechanisms spanning substructure, pair-conditioned, hierarchical, knowledge-graph, transformer, feature-weighting, and contrastive designs.

Attention Type	Ref.	Model	Mechanism	Interpretation and Use
Substructure co-attention	[11]	SSI-DDI	Pairwise fragment weighting	Generates fragment-level hypotheses.
Pair-conditioned attention	[26]	DSN-DDI	Intra-/inter-drug weighting	Separates self-context from pair evidence.
Hierarchical attention	[83]	HAN-DDI	Node and meta-path weighting	Prioritizes biomedical semantic paths.
Adaptive KG attention	[24]	KnowDDI	Edge refinement with relation paths	Produces inspectable evidence trails.
Transformer attention	[23]	DrugDAGT	Local and global molecular attention	Captures long-range dependencies.
Feature weighting	[31]	DMFDDI	Attention-weighted modality fusion	Supports modality-level ablation.
Contrastive attention	[27]	CASE	Dual-view contrastive alignment	Targets imbalance and view mismatch.

**Table 10:** Selected representative fusion strategies spanning bi-level, attention-weighted, orthogonal, contrastive, reconstruction-based, geometric, LLM-enhanced, and multifaceted integration.

Fusion Type	Ref.	Model	Modalities	Methodological Relevance
Bi-level fusion	[87]	MUFFIN	Molecular graph, KG, sequence	Representative multi-level integration.
Attention fusion	[31]	DMFDDI	Graph, network, biochemical features	Tests learned weighting against concatenation.
Orthogonal projection	[36]	MOPDDI	Substructure, target, enzyme, pathway	Reduces cross-modality redundancy.
Domain-invariant fusion	[25]	DSIL-DDI	Substructure-interaction views	Targets unseen-drug generalization.
Masked reconstruction	[32]	KGMAEDDI	Molecular graph and KG	Aligns structured views through reconstruction.
2D/3D fusion	[17]	SGT-DDI	Graph and conformation	Evaluates complementary geometric evidence.
LLM-enhanced fusion	[21]	LLM-DDI	Text embeddings and biomedical KG	Adds text-rich semantic context.
Multifaceted fusion	[33]	Multifaceted model	Text, graphs, images, formulas	Shows that more modalities are not always better.

**Table 11:** Validation and reporting checklist for reducing bias, improving reproducibility, and supporting clinically meaningful interpretation in GNN-based DDI prediction studies.

Item	Why It Matters	Minimum Reporting	Clinical/Translational Relevance
Negative sampling	Unknown pairs may not be true negatives	Sampling rule, ratio, random seed, and sensitivity analysis	Reduces inflated performance from easy artificial negatives [9,22].
Split design	Random pairwise splits can leak drug identity	Pairwise, drug-disjoint, temporal, and/or external split definitions	Matches the intended deployment setting for new drugs or new evidence [8].
Modality ablation	Extra modalities can add redundancy or leakage	Remove each modality and report performance changes	Identifies whether chemical, KG, text, or network evidence drives predictions [33].
Cold-start testing	Many clinical uses involve unseen drugs or sparse labels	New-drug, new-new pair, and rare-class metrics	Prevents overstating usefulness for newly approved or rarely combined drugs [25,26].
Calibration	High scores do not guarantee reliable probabilities	Reliability curves, calibration error, or uncertainty intervals	Supports threshold selection and avoids unsafe overconfident alerts [28].
Interpretability	Attention alone is not causal proof	Attention maps, subgraphs, paths, and ablation validation	Links predictions to enzymes, targets, pathways, or adverse-event mechanisms [24,37].
Reproducibility	Small preprocessing differences can change DDI results	Code, data partitions, hyperparameters, versions, and identifiers	Enables fair comparison and regulatory or hospital review [34,35].
External validation	Benchmarks may not reflect real medication safety use	Cross-database, pharmacovigilance, temporal, or expert-review validation	Provides evidence beyond internal benchmark performance [6,8].

**Table 12:** Computational-cost and scalability considerations for representative GNN-based DDI model families. Exact runtime, memory, and parameter counts were not consistently reported, so entries distinguish reported lightweight evidence from qualitative complexity concerns.

Model Family	Representative Examples	Scalability Implication	Recommended Reporting
Lightweight GAT/KGAT or simple fusion	LaGAT, DDI-KGAT, MDG-DDI	Lower-dimensional embeddings and simpler aggregators are more suitable for resource-constrained screening, but may miss long-range or multimodal mechanisms [63,84,94].	Embedding size, parameter count, inference time per drug pair, and CPU/GPU setting.
Dual-view and contrastive GNNs	MIRACLE, DSN-DDI, DSIL-DDI, MRGCDDI	Additional views and contrastive batches improve representation learning but increase preprocessing and training cost [12,25,26,68].	View-construction time, batch size, augmentation cost, and cold-start inference cost.
Graph transformers and 2D/3D fusion	DrugDAGT, Taco-DDI, SGT-DDI, ALG-DDI	Self-attention and conformer processing can capture global dependencies but may scale poorly with atoms, fragments, tokens, and conformations [16,17,23,77].	Parameter count, maximum graph size, conformer-generation cost, GPU memory, and latency.
Hypergraph and higher-order models	MFHNN-DDI, HCAN	Hyperedge construction can model higher-order relations but adds dataset-specific graph-building overhead [18,59].	Hyperedge rules, graph density, construction time, and sensitivity to sparse labels.
LLM-enhanced fusion	LLM-DDI, DDI-GPT, BioFocal-DDI, KITE-DDI	Text extraction and large embeddings can improve semantic coverage but introduce model-version, retrieval, memory, and reproducibility dependencies [21,52,56,88].	LLM version/checkpoint, prompt set, extraction pipeline, embedding dimension, memory, and update policy.

**Table 13:** Use-case-specific mapping of DDI prediction evidence sources, model choices, and validation designs.

Use Case	Most Relevant Evidence	Suitable Model Choice	Required Validation	Key Risk if Missing
Binary screening	Molecular graph, DDI topology, descriptors	Baseline GAT/GCN plus simple fusion	Random split plus drug-disjoint sensitivity	Overstated gains from memorized drug identity [11,12].
Event-type prediction	Drug-pair labels, adverse events, KG relations	Multi-class GNN, KG model, or attention fusion	Per-class F1, macro-F1, rare-class analysis	High overall accuracy can hide weak rare-event prediction [13,87].
Cold-start drugs	Molecular graphs, targets, pathways, semantic context	Dual-view, contrastive, or inductive HGNN	New-drug and new-new splits	Pairwise splits may not measure generalization [25,26].
Mechanism discovery	Enzymes, transporters, proteins, pathways, KG paths	Heterogeneous GNN or KG attention	Path plausibility, expert review, ablation	Predictions may lack pharmacological explanation [24,37].
Text-rich evidence	Literature, labels, case reports, free-text DDI descriptions	LLM-enhanced fusion with structured graph features	External text-source validation and prompt/version reporting	Semantic extraction errors become hidden model inputs [21,88].
2D/3D molecular reasoning	2D molecular graph, 3D conformation, atom features	Graph transformer or 2D/3D fusion model	Conformation sensitivity and substructure ablation	3D features may add cost without stable benefit [17].
Sparse-label setting	Molecular views, DDI network, KG neighborhoods	Contrastive or self-supervised GNN	Label-scarcity curves and negative-sampling tests	Performance may depend on arbitrary negatives [20,68].
Clinical decision support	Severity, actionability, uncertainty, explanation paths	Calibrated and interpretable multimodal model	Prospective workflow or external hospital testing	High false-alert burden can reduce usability [28].
Pharmacovigilance triage	Temporal reports, external DDI databases, known safety signals	Graph-enhanced ranking or signal-detection model	Temporal validation and signal-review comparison	Retrospective benchmarks may miss reporting bias [8,22].
Deployment monitoring	Versioned data, model outputs, drift indicators	Lightweight monitored model or ensemble	Periodic recalibration and drift assessment	Performance can degrade as drugs and evidence change [34,35].

**Table 14:** Data-modality checklist for constructing DDI prediction inputs.

Modality	What It Adds	Common Preprocessing Risk	Recommended Check
Molecular graph	Atom, bond, ring, and functional-group topology	Inconsistent protonation, salts, tautomers, or atom features	Standardize structures and report graph construction rules [11,30].
SMILES sequence	Complementary sequential chemical patterns	Non-canonical strings and augmentation differences	Specify canonicalization, tokenization, and augmentation policy [56].
Known DDI network	Interaction topology and neighborhood similarity	Leakage when train and test share the same high-degree drugs	Report drug-disjoint and temporal sensitivity tests [12,22].
Knowledge graph	Targets, enzymes, pathways, diseases, adverse events	Missing or biased biomedical relations	Document graph source, relation filtering, and entity mapping [13,24].
Protein/target data	Mechanistic pharmacodynamic or pharmacokinetic context	Incomplete target coverage and synonym mismatch	Harmonize identifiers and separate known from inferred targets [37].
Side-effect profile	Phenotypic similarity between drugs	Confounding by indication or reporting frequency	Use external adverse-event validation where possible [8].
Biomedical text	Mechanistic clues not structured in databases	Extraction errors, prompt sensitivity, and version drift	Record model version, prompts, extraction rules, and manual checks [21,88].
3D conformation	Spatial and stereochemical information	Conformer selection and computational cost	Test conformation sensitivity and compare against 2D-only baselines [17].
Multimodal embedding	Integrated chemical, biological, and semantic evidence	Redundancy, missingness, and hidden leakage	Perform modality ablation and missing-modality stress tests [31,33].
Clinical metadata	Severity, actionability, patient and workflow context	Privacy limits and population bias	Validate prospectively and monitor calibration across subgroups [28].

**Table 15:** Evaluation protocols and reporting requirements for DDI prediction studies [9,22,34,35].

Protocol	What It Tests	Main Bias Risk	Recommended Reporting
Random pairwise split	Interpolation among known drugs and interaction patterns	Same drugs appear in training and test sets, inflating apparent generalization	Report as transductive benchmark only; include drug overlap statistics
Drug-disjoint or cold-start split	Prediction for unseen drugs or unseen drug pairs	Lower performance may reveal dependence on known-drug memorization	Report whether one or both drugs are unseen and provide class-wise results
Temporal split	Prediction of interactions discovered after a cutoff date	Requires accurate evidence timestamps and database-version control	State cutoff dates, database versions, and whether future evidence was excluded from features
External-database validation	Transfer to a different DDI source or benchmark	Differences in curation policy, label type, and drug coverage can confound results	Report identifier mapping, overlap, missing labels, and calibration
Multi-class/event-level evaluation	Mechanism, adverse-event, or severity prediction	Class imbalance and label ambiguity can hide rare-event failure	Report macro-F1, per-class recall, confusion matrices, and rare-class performance
Clinical or pharmacovigilance validation	Utility in medication-safety or signal-detection workflows	Observational bias, confounding, and incomplete outcome capture	Report actionability, calibration, uncertainty, expert review, and prospective feasibility

**Table 16:** Integrated recommendations for model design in GNN-based DDI prediction.

Use Case	Recommended Approach	Key Evidence
Binary, homogeneous features	Concatenation + Basic GAT	MDG-DDI: original inductive evaluation; interpret reported F1 gain only with the same dataset, split, and baselines [63]
Multi-class/multi-label	Attention-weighted/Bi-level fusion	DMFDDI: original multi-type benchmark; compare against the same-split concatenation and single-modality baselines [31]
Cold-start/unseen drugs	Dual-view + Contrastive attention	DSIL-DDI: original unseen-drug split; interpret reported ACC gain only against the paper’s stated baselines [25]
Rich biomedical KGs	Heterogeneous GNN + Hierarchical attention	HAN-DDI: original heterogeneous-graph benchmark; interpret F1 only with the same entity types, split, and baselines [83]
2D+3D/multiscale fusion	Graph Transformer + Cross-attention	SGT-DDI: original 2D/3D fusion benchmark; compare only against same-split 2D-only, 3D-only, and fusion baselines [17]
Higher-order relationships	Hypergraph + Multi-level fusion	MFHNN-DDI: original hypergraph benchmark; interpret accuracy with the reported hyperedge construction, split, and baselines [18]
Unstructured text available	LLM-enhanced fusion	DDI-GPT: original new-FDA zero-shot evaluation; interpret reported AUROC gain only with the same comparator, prompt/version, and split controls [88]
Low-data/imbalanced	Contrastive learning	CASE: robust at 1:3 imbalance [27]
Unknown optimal architecture	Automated GNAS (AutoDDI)	5–8 layers optimal [81]
Resource-constrained deployment	Lightweight (32-dimensional, small memory)	DDI-KGAT: 32-dimensional [84]
Clinical decision support	Calibrated score + evidence trail	KnowDDI paths and SGT-DDI attention maps are hypotheses requiring validation [17,24]
Redundant modalities	Ablation-driven selection	The trimodal configuration outperformed the five-modality configuration [33].

**Table 17:** Practical distinctions among DDI-related resources for clinicians.

Resource Type	Typical Access	Appropriate Use	Main Caution
Institutional prescribing or pharmacy checker	Institution-dependent	Routine medication-safety screening	Follow local policy and alert-management procedures
Open-access curated resource	Public web access	Review documented interactions and supporting information	Coverage and update dates vary
Subscription drug-information resource	Account, institutional access, or commercial license may be required	Detailed evidence, mechanisms, and management guidance	Verify access terms and the current resource version
Research GNN model or demonstration	Research access; technical setup may be required	Reproduction, benchmarking, and hypothesis generation	Not independently sufficient for treatment decisions

**Table 18:** Clinical translation pathway for GNN-based DDI prediction systems [24,28,37].

Stage	Potential Use	Evidence Needed Before Use	Key Safeguard
Benchmark research	Compare graph representations, fusion strategies, and attention mechanisms	Transparent splits, baselines, ablations, and reproducible code	Treat results as methodological evidence, not clinical proof.
Drug-development triage	Prioritize risky combinations for laboratory or pharmacology review	External validation, mechanistic plausibility, and uncertainty estimates	Require expert review before changing development decisions.
Pharmacovigilance signal detection	Rank possible DDIs in spontaneous reports or real-world evidence	Temporal validation, calibration, and comparison with known safety signals	Control for reporting bias and confounding.
Clinical decision support	Flag high-risk combinations at prescribing or medication review	Prospective workflow testing, severity/actionability labels, and false-alert analysis	Present evidence trails and avoid alert fatigue.
Regulatory or hospital governance	Support surveillance, audit, and model monitoring	Documentation of data provenance, fairness, versioning, and post-deployment drift	Maintain human oversight and periodic revalidation.

## Data Availability

No new data or predictive models were generated. Extracted study-level data are provided in the manuscript and Supplementary Materials, and supporting extraction records are available from the corresponding authors upon reasonable request. No custom analytical code was used.

## Supplementary Materials

The following supporting information can be downloaded at https://www.mdpi.com/article/10.3390/ph19081225/s1, Table S1: Completed PRISMA 2020 checklist and manuscript location for the systematic review; Table S2: Study-level inventory of datasets and source-checked evaluation metrics for graph-based or graph-enhanced DDI studies; Table S3: Eligibility, evidence-extraction, and review-decision rules used in the systematic review; Table S4: Aggregate study-selection accounting underlying the PRISMA flow diagram; Table S5: Aggregate audit of data-leakage, uncertainty, reproducibility, and computational-resource reporting across the 60 included studies; Table S6: Advantages and limitations of major GNN architecture families for DDI prediction [11,18,23,26,81]; Table S7: Integrated synthesis of GNN architecture, attention mechanism, and multimodal-fusion choices; Table S8: Extended inventory of heterogeneous, knowledge-graph, transformer, contrastive, and architecture-search GNN approaches for DDI prediction; Table S9: Extended inventory of hierarchical, knowledge-graph, transformer, feature-weighting, and contrastive attention mechanisms for DDI prediction; Table S10: Extended inventory of LLM-enhanced, hypergraph, contrastive, and knowledge-adaptive fusion strate-gies for DDI prediction. References [110,111,112] are in Supplementary Materials.

## Abbreviations

The following abbreviations are used in this manuscript:

API	Application programming interface
AUC	Area under the curve
AUPR	Area under the precision–recall curve
AUROC	Area under the receiver operating characteristic curve
CYP	Cytochrome P450
DDI	Drug–drug interaction
FDA	U.S. Food and Drug Administration
GAT	Graph attention network
GCN	Graph convolutional network
GNN	Graph neural network
GPU	Graphics processing unit
IoMT	Internet of Medical Things
KEGG	Kyoto Encyclopedia of Genes and Genomes
KGAT	Knowledge graph attention network
LLM	Large language model
MPNN	Message-passing neural network
PRISMA	Preferred Reporting Items for Systematic Reviews and Meta-Analyses
SMILES	Simplified molecular-input line-entry system
