# Supplementary material for: Graph-Based Machine Learning for Predicting Drug–Drug Interactions: A Systematic Review
Source: Pharmaceuticals (Basel). 2026 Aug 4;19(8):1225. doi: 10.3390/ph19081225 (PMC13516268; doi:10.3390/ph19081225)
Supplement: Supplementary file 1 [file pharmaceuticals-19-01225-s001.zip › pharmaceuticals-4420127-supplementary.pdf]

# Supplementary Material

## Graph-Based Machine Learning for Predicting Drug–Drug Interactions: A Systematic Review

Md. Tuhin Reza, Md. Abdul Kader, Wissem Inoubli, Md. Biddut Hossain, and Md. Kamrul Islam

Table S1: Completed PRISMA 2020 checklist and manuscript location for the systematic review.

| Item | Section | Checklist Requirement Addressed in this Manuscript | Location/Status |
|---|---|---|---|
| 1 | Title | Identifies the report as a systematic review. | Title |
| 2 | Abstract | Provides structured background/objectives, methods, results, and conclusions. | Abstract |
| 3 | Rationale | Explains why a focused review of graph-based DDI prediction is needed. | Introduction |
| 4 | Objectives | States the review question and the focus on GNN architecture, multimodal fusion, attention, validation, and interpretability. | Introduction; Review Methodology |
| 5 | Eligibility Criteria | Specifies eligible and excluded study types, model scope, empirical-evaluation requirements, and graph-based versus graph-enhanced definitions. | Eligibility Criteria |
| 6 | Information Sources | Lists databases, date limits, final search update, and citation-tracing use. | PRISMA Framework; main-manuscript Table 1 |
| 7 | Search Strategy | Reports database-specific search terms, filters, and record counts. | Main-manuscript Table 1 |
| 8 | Selection Process | Describes title/abstract screening, full-text eligibility assessment, screening by at least two authors, consensus resolution, and absence of automated eligibility tools. | PRISMA Framework; Eligibility Criteria; main-manuscript Figure 3 |
| 9 | Data Collection Process | Describes structured data extraction, extraction or verification by at least two authors, consensus resolution, no investigator contact, and absence of automated extraction tools. | Data Collection Process and Data Items |
| 10 | Data Items | Lists extracted study characteristics, graph/model variables, validation details, metrics, ablations, interpretability, and availability items; missing information was not imputed. | Data Collection Process and Data Items |
| 11 | Study Risk of Bias | Describes the domain-based risk-of-bias and applicability framework, assessment by at least two authors, consensus resolution, and absence of automated risk-of-bias tools. | Risk-of-Bias and Applicability Assessment; main-manuscript Table 2 |
| 12 | Effect Measures | States that no clinical effect measures were defined and that benchmark metrics were extracted as originally reported. | Data Collection Process and Data Items; Synthesis Methods |
| 13 | Synthesis Methods | Explains why meta-analysis was not performed and describes narrative grouping, comparative dimensions, and treatment of within-study performance claims. | Synthesis Methods; Results (Sections 3.1–3.6); Discussion and Clinical Interpretation |
| 14 | Reporting Bias Assessment | States that formal statistical publication-bias assessment was not appropriate and describes qualitative consideration of reporting completeness. | Risk-of-Bias and Applicability Assessment; Limitations |
| 15 | Certainty Assessment | States that GRADE was not used and explains narrative confidence assessment based on reproducibility and validation strength. | Risk-of-Bias and Applicability Assessment; Conclusions |
| 16 | Study Selection | Provides counts for records identified, duplicates removed, records screened, reports sought and assessed, exclusions, and included studies. | PRISMA Framework; main-manuscript Figure 3 |
| 17 | Study Characteristics | Summarizes datasets, model families, modalities, validation protocols, and representative study-level evidence. | Results (Sections 3.1–3.6); Discussion and Clinical Interpretation; Supplementary Tables S2 and S6–S10 |
| 18 | Risk of Bias in Studies | Summarizes study-level risk/applicability groups and key bias domains such as leakage, negative sampling, weak external validation, limited reproducibility, and unvalidated interpretability. | Main-manuscript Tables 2 and 3; Sections 4.4, 4.6, and 4.9 |
| 19 | Results of Individual Studies | Reports representative study-level datasets, metrics, and model claims without treating them as pooled effects. | Results; Section 4.7; Supplementary Tables S2 and S8–S10 |
| 20 | Results of Syntheses | Presents narrative syntheses across model families, attention mechanisms, fusion strategies, validation protocols, scalability, and clinical translation. | Results (Sections 3.1–3.6); Discussion and Clinical Interpretation (Sections 4.1–4.9) |
| 21 | Reporting Biases | Discusses possible selective reporting, incomplete ablation, missing code, and lack of external or temporal validation as limitations. | Sections 4.4, 4.6, and 4.9; Limitations of this Review |
| 22 | Certainty of Evidence | Interprets findings as methodological rather than clinical-certainty claims because benchmarks and validation designs are heterogeneous. | Sections 4.7–4.9; Conclusions |
| 23 | Discussion | Summarizes principal findings, limitations of evidence, interpretation for computational DDI prediction, and implications for future work. | Discussion and Clinical Interpretation; Future Research Directions; Limitations of this Review; Conclusions |
| 24 | Registration and Protocol | States that the review was not prospectively registered, no separate protocol was published, and no protocol amendments were made. | PRISMA Framework |
| 25 | Support | Reports funding status. | Funding |
| 26 | Competing Interests | Reports conflicts of interest. | Conflicts of Interest |

*Continued on next page*

Table S1: Completed PRISMA 2020 checklist and manuscript location for the systematic review (continued).

| Item | Section | Checklist Requirement Addressed in this Manuscript | Location/Status |
|---|---|---|---|
| 27 | Availability of Data, Code, and Materials | States that no new experimental or patient-level data and no model were generated; extracted study-level data are summarized in the manuscript and supplementary tables; the complete evidence-extraction matrix and supporting notes are available from the corresponding authors upon reasonable request; and no custom analytic code was used. | Data Availability Statement |

Table S2: Study-level inventory of datasets and source-checked evaluation metrics for graph-based or graph-enhanced DDI studies. Values are study-specific and do not imply comparability across datasets, tasks, or validation protocols.

| No. | Model / Study | Reference | Dataset(s) or Data Source(s) | Verified Metric / Value |
|---|---|---|---|---|
| 1 | SSI-DDI | [11] | DrugBank DDI event-type benchmark | Transductive ACC 94.47% |
| 2 | Taco-DDI | [16] | Multi-type DDI event benchmark datasets | ACC 91.51% |
| 3 | SGT-DDI | [17] | DrugBank/TWOSIDES-style molecular benchmarks; 2D/3D molecular inputs | DrugBank ACC 97.23% |
| 4 | MFHNN-DDI | [18] | DrugBank-style hypergraph DDI benchmark | DrugBank ACC 98.92% |
| 5 | FG-DDI | [19] | Molecular-graph DDI benchmark datasets | DrugBank transductive ACC 97.30% |
| 6 | KA-DDI | [20] | DrugBank and KEGG interaction networks | DrugBank ACC 83.76% |
| 7 | LLM-DDI | [21] | Biomedical knowledge graph with text-derived features | OGB-biokg AUC 99.44% |
| 8 | DMFDDI | [31] | DrugBank, KEGG, and biochemical feature resources | ZhangDDI F1 97.41% |
| 9 | KGMAEDDI | [32] | Molecular graph plus knowledge-graph DDI benchmarks | Binary ACC 97.71% |
| 10 | Multifaceted fusion model | [33] | Text, image, formula, and graph DDI extraction resources | Micro-F1 84.45% |
| 11 | SA-DDI | [30] | DrugBank DDI event-type benchmark | DrugBank warm ACC 96.23% |
| 12 | SynCaps-DDI | [38] | Biomedical text DDI extraction corpus | DDI-2013 F1 85.44% |
| 13 | ImageDDI | [39] | Molecular motif/image-enhanced DDI benchmark | Ryu's ACC 96.54% |
| 14 | KnowDDI | [24] | DrugBank-derived DDI knowledge subgraphs | TWOSIDES AUROC 95.43% |
| 15 | Multimodal KG-DNN | [40] | Knowledge-graph DDI event benchmark | F1 84.22% |
| 16 | MRLF-DDI | [41] | Multi-view DDI event benchmark datasets | Ryu's ACC 97.05% |
| 17 | MEDL-DDI | [42] | Multi-source DDI benchmark resources | DDInter1.0 Macro-F1 91.5% |
| 18 | KGDB-DDI | [43] | Knowledge graph and drug-background data resources | DrugBank AUC 99.52% |
| 19 | RCAN-DDI | [44] | Drug-pair DDI benchmark datasets | Multi-class ACC 97.67% |
| 20 | AI-DDI | [45] | Substructure-based DDI benchmark datasets | Transductive ACC 95.12% |
| 21 | SCATrans | [46] | Multimodal biomedical DDI benchmark resources | Dataset-2 F1 74.79% |
| 22 | DrugDAGT | [23] | Molecular graph DDI benchmark datasets | Warm-start ACC 99.74% |
| 23 | GMIA | [47] | chch-miner and DeepDDI-style benchmarks | ChCh-Miner AUC 98.12% |
| 24 | DGNN-DDI | [48] | DrugBank/TWOSIDES-style DDI benchmarks | DrugBank ACC 96.09% |
| 25 | DDI-MuG | [49] | Multi-aspect DDI extraction/graph resources | F1 84.7% |
| 26 | SRR-DDI | [50] | DrugBank/TWOSIDES-style molecular DDI benchmarks | DrugBank warm ACC 96.67% |
| 27 | IIB-DDI | [51] | Out-of-distribution DDI benchmark splits | DrugBank transductive ACC 97.02% |
| 28 | DSIL-DDI | [25] | Unseen-drug DDI benchmark splits | DrugBank transductive ACC 98.12% |
| 29 | DSN-DDI | [26] | DrugBank, TWOSIDES, and FDA-approved-drug split | DrugBank transductive ACC 96.94% |
| 30 | BioFocal-DDI | [52] | Biomedical DDI extraction/text benchmark | F1 86.64% |
| 31 | MOPDDI | [36] | Multimodal DDI event benchmark with target/enzyme/pathway data | Dataset 1, Task 1 ACC 95.00% |
| 32 | CASE | [27] | DrugBank and TWOSIDES polypharmacy benchmarks | ACC 88.70% |
| 33 | Memory interaction NN | [53] | Multi-label DDI benchmark datasets | KEGG dataset: 0.8407; OGB-biokg dataset: 0.9878 |
| 34 | EDDINet | [54] | Multi-graph DDI benchmark resources | TWOSIDES AUROC 0.9481 |
| 35 | MMDDI-SSE | [55] | Multi-modal event-type DDI benchmark datasets | Deng ACC 0.9515 |
| 36 | KITE-DDI | [56] | DRKG embeddings with DDI event benchmarks | ACC 95.67% |
| 37 | HDN-DDI | [57] | DrugBank and TWOSIDES benchmarks | TWOSIDES warm ACC 99.38% |
| 38 | Spatial-aware KG capsule model | [58] | Biomedical knowledge graph DDI benchmark | OGB-biokg AUC 0.9928 |
| 39 | Contrastive hypergraph model | [59] | Low-frequency DDI discovery/risk benchmark | ChCh-Miner ACC 0.7961 |
| 40 | HLN-DDI | [60] | DrugBank molecular DDI benchmark | Transductive ACC 98.15% |
| 41 | 3D elastic MPNN model | [61] | 3D molecular DDI benchmark datasets | ZhangDDI ACC 98.61% |
| 42 | HGNN-DDI | [62] | Heterogeneous information network DDI benchmark | 6-class F1 96.91% |
| 43 | MDG-DDI | [63] | DrugBank and related benchmark DDI datasets | DrugBank F1 0.9431 |
| 44 | MGDDI | [64] | Multi-type DDI event benchmark datasets | DrugBank ACC 96.04% |
| 45 | Multi-view contrastive model | [65] | DDI event-prediction benchmark datasets | Deng ACC 95.28% |

Continued on next page

Table S2: Study-level inventory of datasets and source-checked evaluation metrics for graph-based or graph-enhanced DDI studies (continued).

| No. | Model / Study | Reference | Dataset(s) or Data Source(s) | Verified Metric / Value |
|---|---|---|---|---|
| 46 | MMFF-DDI | [66] | Multi-modal, multi-granularity DDI benchmark resources | DeepDDI F1 95.12% |
| 47 | Hierarchical multi-relational model | [67] | Large-scale multi-relational DDI graph benchmark | Dataset 1, Task 1 AUC 99.94% |
| 48 | GCN collaborative-filtering model | [78] | Drug–drug interaction matrix/graph benchmark | Precision@10 = 0.847 |
| 49 | ALG-DDI | [77] | Multiscale molecular and knowledge-graph DDI evidence | DS2 AUROC 99.85% |
| 50 | MRGCDDI | [68] | Multi-relation DDI event benchmarks | Ryu's ACC 96.43% |
| 51 | DualC | [69] | Dual latent-feature DDI benchmark datasets | ACC 97.25% |
| 52 | Multi-dimensional interaction network | [79] | Multi-dimensional DDI network benchmark | DrugBank AUPRC 99.01% |
| 53 | MSHGCL | [70] | Multiscale graph DDI benchmark datasets | DrugBank ACC 96.88% |
| 54 | HTCL-DDI | [71] | Hierarchical triple-view DDI benchmarks | ZhangDDI AUROC 98.58% |
| 55 | MAVGAE | [72] | Asymmetric DDI benchmark datasets | ACC 91.3% (asymmetric task) |
| 56 | Multi-view interpretable model | [73] | Multi-view DDI benchmark resources | BIOSNAP ROC-AUC 93.74% |
| 57 | Interpretable graph model | [37] | Molecular and DDI-network benchmark datasets | Ryu's 5-fold CV ACC 95.2% |
| 58 | MPHGCL-DDI | [74] | Meta-path heterogeneous graph DDI benchmark | Dataset 1, Task 1 Macro-F1 91.07% |
| 59 | SSF-DDI | [75] | DrugBank and TWOSIDES benchmarks | DrugBank transductive ACC 96.45% |
| 60 | KG contrastive method | [76] | Knowledge-graph-based DDI benchmark | Multi-class ACC 98.01% |

Table S3: Eligibility, evidence-extraction, and review-decision rules used in the systematic review.

| Domain | Operational Rule or Extracted Information | Review Handling |
|---|---|---|
| Eligible DDI tasks | Prediction, event classification, graph-supported extraction, or prioritization of drug–drug interactions. | Retained under the umbrella term DDI prediction while preserving the task distinction in evidence tables. |
| Eligible graph methods | GNNs, graph-derived neural representations, biomedical knowledge graphs, molecular graphs, DDI networks, hypergraphs, or graph-enhanced multimodal models. | The graph component had to contribute directly to the DDI pipeline. |
| Empirical evidence | Benchmark datasets, curated biomedical resources, or clinically relevant DDI labels with reported model evaluation. | Studies without empirical evaluation were excluded. |
| Minimum reporting detail | Data source, graph construction, model architecture, and evaluation metric had to be identifiable. | Reports with insufficient methodological detail were excluded. |
| Graph-based definition | A molecular graph, DDI network, knowledge graph, or hypergraph served as the primary backbone for message passing or graph reasoning. | Included as a primary graph-based study. |
| Graph-enhanced definition | Graph-derived features or knowledge-graph paths were included within a multimodal or LLM-enhanced pipeline. | Included only when the graph contribution was identifiable in the architecture or inputs. |
| Principal exclusions | Non-DDI tasks, traditional non-graph machine learning, unimodal methods without a graph component, absence of empirical evaluation, or insufficient detail. | No otherwise eligible report was excluded because of low performance or unfavorable results. |
| Screening process | Titles/abstracts and retrieved full texts were assessed by at least two authors. | Screening was parallel rather than blinded; disagreements were resolved by consensus. |
| Automation and contact | No automated eligibility or extraction tools were used; study investigators were not contacted. | Published reports were the source of all extracted synthesis variables. |
| Study characteristics | Publication year, task, graph type, node/edge definitions, data sources, modalities, and benchmark dataset. | Recorded in the structured evidence matrix. |
| Model characteristics | GNN architecture, message passing, attention, fusion, ablations, and interpretability method. | Preserved using harmonized terminology without altering the original study claims. |
| Validation characteristics | Split design, negative sampling, metrics, comparators, external/temporal testing, and generalization setting. | The primary task and the setting most relevant to generalization were emphasized. |
| Reproducibility characteristics | Code/data availability, preprocessing information, framework, hyperparameters, hardware, and reported limitations. | Missing or unclear information was recorded as not reported rather than imputed. |
| Outcome handling | AUROC, AUPR, F1 score, accuracy, precision, recall, and related benchmark metrics. | Metrics were retained as reported; no cross-study transformation or pooled effect calculation was performed. |
| Risk/applicability domains | Leakage, negative sampling, generalization, baseline/ablation adequacy, reproducibility, interpretability, and clinical applicability. | Domains informed an overall concern grouping and narrative assessment rather than a numerical score. |

Table S4: Aggregate study-selection accounting underlying the PRISMA flow diagram.

| **Selection Stage** | **Count** | **Explanation** |
|---|---|---|
| Database records identified | 290 | IEEE Xplore 74; ScienceDirect 96; PubMed 67; Google Scholar 53. |
| Duplicate records removed | 108 | Duplicate database records and overlapping indexed versions. |
| Records removed before screening | 12 | Failed language, document-type, date, or computational-DDI scope requirements based on bibliographic metadata. |
| Titles and abstracts screened | 170 | Records remaining after deduplication and pre-screening. |
| Reports sought for retrieval | 111 | Records considered potentially eligible after title/abstract screening. |
| Reports not retrieved | 18 | Inaccessible full texts, duplicate conference/journal versions where the fuller report was retained, or insufficient bibliographic information. |
| Full-text reports assessed | 93 | Retrieved reports evaluated against the complete eligibility criteria. |
| Excluded: no GNN or graph-based neural method | 17 | Did not satisfy the primary graph-method eligibility criterion. |
| Excluded: unimodal or outside focused graph-enhanced scope | 16 | Did not satisfy the defined graph-enhanced synthesis scope. |
| Primary studies included | 60 | Final evidence base used for the study-level synthesis. |

Table S5: Aggregate audit of data-leakage, uncertainty, reproducibility, and computational-resource reporting across the 60 included studies.

| **Reporting Item** | **Count** | **Percentage** | **Interpretation or Reported Range** |
|---|---|---|---|
| Data leakage mentioned or addressed | 3 | 5.0% | P007: LLM-DDI (Li et al., 2025); P008: DMFDDI (Gan et al., 2023); P015: Multimodal KG-DNN (Shen et al., 2025). |
| No explicit data-leakage discussion | 57 | 95.0% | No explicit discussion of data leakage was identified. |
| Mentioned uncertainty or calibration | 4 | 6.7% | Any explicit discussion of predictive uncertainty or calibration. |
| No uncertainty discussion | 56 | 93.3% | No explicit uncertainty or calibration assessment. |
| Probability-calibration method | 2 | 3.3% | Included a reported probability-calibration approach. |
| Confidence scores | 2 | 3.3% | Reported confidence scores associated with predictions. |
| Bayesian or probabilistic method | 3 | 5.0% | Used Bayesian, variational, or another probabilistic approach. |
| Ensemble-based uncertainty | 1 | 1.7% | Used ensemble outputs to characterize uncertainty. |
| Code available | 31 | 51.7% | Public or otherwise reported code availability. |
| Code unavailable | 29 | 48.3% | No accessible code was reported. |
| Implementation framework specified | 60 | 100.0% | A software or deep-learning framework was named. |
| At least some hyperparameters reported | 59 | 98.3% | Partial hyperparameter information was available. |
| All key hyperparameters reported | 38 | 63.3% | Sufficient key settings were reported for closer reconstruction. |
| GPU specified | 8 | 13.3% | Examples included A100, V100, RTX 3090, RTX 2080 Ti, A40, and RTX 2070 Ti. |
| Training time reported | 12 | 20.0% | Reported values ranged from approximately 35 s/epoch to 5 hours. |
| Inference time reported | 6 | 10.0% | Reported values ranged from approximately 1 ms to 8–10 ms per sample. |
| Parameter count reported | 18 | 30.0% | Reported model sizes ranged from approximately 4.1 million to 120 million parameters. |
| Memory usage reported | 8 | 13.3% | Reported memory requirements ranged from approximately 4 GB to 9.5 GB. |
| Scalability analysis | 15 | 25.0% | Addressed large datasets, limited training data, efficiency, or related scalability considerations. |

*Note:* The two data-leakage reporting categories are mutually exclusive and exhaustive. They describe whether leakage was explicitly discussed, not whether leakage was definitively present or absent. Uncertainty-method categories are not mutually exclusive; a study could contribute to more than one method category. All percentages use 60 unique included studies as the denominator. This audit supports the reporting-frequency results in Section 3.5 and the discussions of calibration and reproducibility (Section 4.4), computational cost (Section 4.5), and data leakage (Section 4.9.2) in the main manuscript.

# Detailed Architecture, Attention, and Fusion Tables Moved from the Main Manuscript

The following tables preserve the detailed comparisons removed from the main manuscript during condensation. They extend the architecture, attention, fusion, and integrated syntheses in Sections 3.2–3.4 and 3.6, the corresponding interpretation in Sections 4.1–4.3, and the external-validation discussion in Section 4.6. They should be interpreted together with the complete study-level inventory in Supplementary Table S2, which supports the study-level performance discussion in Section 4.7.

Table S6: Advantages and limitations of major GNN architecture families for DDI prediction [11, 18, 23, 26, 81].

| **Architecture** | **Key Advantages** | **Key Disadvantages** |
|---|---|---|
| Basic GAT/GCN | Interpretable attention; depth can be stabilized with gating or attention | Drug order sensitivity; cold-start performance drop |
| Dual-View/Multiscale | Complementary intra-/inter-drug information; inductive gains | High complexity; dataset-specific tuning |
| Heterogeneous/KG | Rich biomedical context; potential true inductive capability | KG quality dependency; high computational cost |
| Graph Transformers | Global dependency capture; 2D/3D and cross-modal fusion | Large parameter counts; data-hungry |
| Hypergraph | Higher-order relationships; rare-event and calibrated modeling potential | Scalability challenges; decomposition and modality limitations |
| Contrastive | Data-scarcity mitigation; domain-invariant representation learning | Negative sampling sensitivity; many models remain binary-only |
| Automated GNAS | Dataset-specific architecture discovery; deeper GNN exploration | Computationally expensive; search-space design expertise needed |

Table S7: Integrated synthesis of GNN architecture, attention mechanism, and multimodal-fusion choices.

| **Component** | **Critical Contribution** | **Common Failure Mode** | **Practical Implication** |
|---|---|---|---|
| GNN architecture | Encodes the main biomedical structure: molecular graphs for atom–bond chemistry, DDI networks for interaction topology, heterogeneous KGs for mechanistic context, and hypergraphs for higher-order relations. | Graph choice can dominate results; random pairwise splits may reward memorization of drug topology, while incomplete KGs or noisy DDI networks can propagate biased labels. | Choose the graph type according to the pharmacological question and report drug-disjoint, temporal, or external validation when claiming generalization [11, 22, 24, 26]. |
| Attention mechanism | Provides adaptive weighting over atoms, bonds, substructures, neighbors, relations, layers, or modalities, enabling pair-specific emphasis and some interpretability. | Attention weights can be mistaken for causal explanations; cross-attention and co-attention can be computationally expensive and sensitive to drug order or redundant substructures. | Use attention to guide hypothesis generation, but support claims with ablation, case studies, and pharmacological validation rather than visualization alone [16, 17, 23, 30]. |
| Multimodal fusion | Combines complementary chemical, biological, network, adverse-event, text, and knowledge-graph evidence to improve robustness and mechanistic coverage. | Over-fusion can add redundant or leaking modalities, increase missing-modality sensitivity, and obscure which evidence source drives a prediction. | Prefer parsimonious fusion; report modality ablations, missing-modality stress tests, external knowledge provenance, and leakage safeguards [17, 31–33]. |
| Integrated design | Aligns graph backbone, attention module, and fusion layer so that each component contributes distinct evidence rather than duplicating the same signal. | Highly complex hybrids may improve benchmark scores while reducing reproducibility, calibration, interpretability, and clinical actionability. | Treat accuracy as only one criterion; evaluate calibration, uncertainty, rare-class performance, computational cost, reproducibility, and clinical plausibility [6, 8, 9, 28]. |

Table S8: Extended inventory of heterogeneous, knowledge-graph, transformer, contrastive, and architecture-search GNN approaches for DDI prediction.

| Family | Ref. | Model | Main Design | Methodological Relevance |
|---|---|---|---|---|
| Heterogeneous attention | [83] | HAN-DDI | Entity and meta-path attention | Highlights that meta-path choice can be as important as node-level aggregation. |
| Meta-path contrastive | [74] | MPHGCL-DDI | HGNN + contrastive views | Links heterogeneous biomedical semantics with contrastive cold-start learning. |
| Inductive HGNN | [110] | ACDGNN | Cross-domain transformation | Avoids aggregating DDI edges directly, supporting stronger unseen-drug claims. |
| Pre-trained HGNN | [13] | HetDDI | Atom masking + KG link prediction | Shows how pre-training can reduce dependence on limited DDI labels. |
| KG attention | [84] | DDI-KGAT | KGAT with simple aggregators | Demonstrates that lightweight KG embeddings can still be competitive. |
| Knowledge subgraphs | [24] | KnowDDI | Adaptive edge addition/removal | Adds explanatory relation paths and compensates for sparse or missing KG edges. |
| Unified drug-pair graph | [37] | MolecBioNet | CASPool + AGIPool | Treats a drug pair as a context-dependent entity rather than two isolated molecules. |
| Multi-relational graph | [67] | HMGRL | RGCN + differentiable clustering | Captures implicit relation clusters without external post-processing. |
| Multi-channel KG | [111] | MKG-FENN | Four knowledge graphs + fusion | Illustrates how separate biomedical graph channels can preserve complementary evidence. |
| Graph transformer | [23] | DrugDAGT | Bond and atom dual attention | Combines local message passing with global self-attention and structural biases. |
| Dynamic co-attention | [16] | Taco-DDI | Co-attention-guided graph Transformer | Updates pair-specific attention across transformer layers. |
| 2D/3D transformer | [17] | SGT-DDI | 2D graph + 3D conformation | Shows that conformational evidence can complement two-dimensional topology. |
| Knowledge-adaptive CL | [20] | KA-DDI | KAGAT + contrastive sampling | Addresses interaction dominance and knowledge overload in sparse and dense networks. |
| Automated search | [81] | AutoDDI | Graph neural architecture search | Shows that optimal GNN depth and operator choice are dataset dependent. |

Table S9: Extended inventory of hierarchical, knowledge-graph, transformer, feature-weighting, and contrastive attention mechanisms for DDI prediction.

| Attention Type | Ref. | Model | Mechanism | Interpretation and Use |
|---|---|---|---|---|
| Hierarchical attention | [83] | HAN-DDI | Node-level + meta-path-level | Prioritizes both entities and biomedical semantic paths. |
| Meta-path attention | [74] | MPHGCL-DDI | Attention over heterogeneous paths | Supports cold-start reasoning through protein and disease context. |
| KG attention | [84] | DDI-KGAT | KG neighbor weighting | Lightweight relation prioritization in knowledge graphs. |
| Adaptive subgraph attention | [24] | KnowDDI | Edge pruning/addition with paths | Produces evidence trails that can be inspected pharmacologically. |
| Masked-autoencoder attention | [32] | KGMAEDDI | KG–molecule cross-attention | Aligns molecular and KG views through reconstruction. |
| 2D/3D self-attention | [17] | SGT-DDI | Multi-head molecular transformer | Highlights atomic groups while integrating conformational context. |
| Feature weighting | [31] | DMFDDI | Attention-weighted fusion | Learns modality importance rather than assuming equal weights. |
| Dimension-level attention | [85] | DANN-DDI | Element-wise product attention | Emphasizes informative feature dimensions in drug-pair representations. |
| Squeeze-excitation | [86] | MMADL | Channel attention | Reweights multiple molecular and network channels. |
| Contrastive attention | [27] | CASE | InfoNCE dual-view alignment | Improves robustness when class imbalance or view mismatch is present. |

Table S10: Extended inventory of LLM-enhanced, hypergraph, contrastive, and knowledge-adaptive fusion strategies for DDI prediction.

| Fusion Type | Ref. | Model | Modalities | Methodological Relevance |
|---|---|---|---|---|
| LLM + KG | [21] | LLM-DDI | Text embeddings + biomedical KG | LLM-derived semantic features can stabilize text-rich DDI prediction. |
| Sentence-tree KG | [88] | DDI-GPT | KG paths converted to text | Supports zero-shot reasoning but still needs biological validation. |
| BioGPT augmentation | [52] | BioFocal-DDI | Text, sequence, graph features | Text generation can help minority classes but depends on source quality. |
| Structured extraction | [89] | Multi-feature ML | GPT extraction + curated features | Highlights the role of structured extraction from unstructured DDI descriptions. |
| SMILES + KG transformer | [56] | KITE-DDI | SMILES transformer + DRKG embeddings | Combines sequence and KG embeddings with deployment-oriented memory constraints. |
| Multifaceted fusion | [33] | Multifaceted model | Text, graphs, images, formulas | Demonstrates that more modalities are not always better; ablation is necessary. |
| Hypergraph fusion | [18] | MFHNN-DDI | Hypergraph + GCN + Conv1D | Restores the higher-order fragment relationship perspective. |
| Contrastive perturbation | [68] | MRGCDDI | Molecular + event graphs | Avoids augmentation that may change molecular semantics. |
| Subgraph contrastive | [112] | DeepGCL | Molecular structure + DDI topology | Aligns local subgraph evidence with network-level interaction context. |
| Knowledge-adaptive fusion | [20] | KA-DDI | Molecular, interaction, KG views | Balances interaction dominance against knowledge overload. |

# References


[1] Edwards, I.R.; Aronson, J.K. Adverse drug reactions: definitions, diagnosis, and management. *The lancet* **2000**, *356*, 1255–1259.

[2] Makiani, M.J.; Nasiripour, S.; Hosseini, M.; Mahbubi, A. Drug-drug interactions: The importance of medication reconciliation. *Journal of research in pharmacy practice* **2017**, *6*, 61–62.

[3] Hammoud, A.; Shapiro, M.D. Drug interactions: What are important drug interactions for the most commonly used medications in preventive cardiology? *The Medical Clinics of North America* **2022**, *106*, 389–399.

[4] Stopfer, P. Drug–Drug Interaction Studies. In *Drug Discovery and Evaluation: Methods in Clinical Pharmacology*; Springer, 2020; pp. 827–846.

[5] Zhao, Y.; Yin, J.; Zhang, L.; Zhang, Y.; Chen, X. Drug–drug interaction prediction: databases, web servers and computational models. *Briefings in Bioinformatics* **2024**, *25*, bbad445.

[6] Wang, N.N.; Zhu, B.; Li, X.L.; Liu, S.; Shi, J.Y.; Cao, D.S. Comprehensive review of drug–drug interaction prediction based on machine learning: current status, challenges, and opportunities. *Journal of chemical information and modeling* **2023**, *64*, 96–109.

[7] Zhang, Y.; Deng, Z.; Xu, X.; Feng, Y.; Junliang, S. Application of artificial intelligence in drug–drug interactions prediction: a review. *Journal of chemical information and modeling* **2023**, *64*, 2158–2173.

[8] Xia, Y.; Xiong, A.; Zhang, Z.; Zou, Q.; Cui, F. A comprehensive review of deep learning-based approaches for drug–drug interaction prediction. *Briefings in Functional Genomics* **2025**, *24*, elae052.

[9] Ru, X.; Li, Z.; Wei, L.; Liu, Y.; Zou, Q. Drug–Drug Interaction Prediction: Paradigm Shifts, Key Bottlenecks, and Future Directions. *Wiley Interdisciplinary Reviews: Computational Molecular Science* **2025**, *15*, e70056.

[10] Yu, Y.; Huang, K.; Zhang, C.; Glass, L.M.; Sun, J.; Xiao, C. SumGNN: multi-typed drug interaction prediction via efficient knowledge graph summarization. *Bioinformatics* **2021**, *37*, 2988–2995.

[11] Nyamabo, A.K.; Yu, H.; Shi, J.Y. SSI–DDI: substructure–substructure interactions for drug–drug interaction prediction. *Briefings in Bioinformatics* **2021**, *22*, bbab133.

[12] Wang, Y.; Min, Y.; Chen, X.; Wu, J. Multi-view graph contrastive representation learning for drug-drug interaction prediction. In Proceedings of the Proceedings of the web conference 2021, 2021, pp. 2921–2933.

[13] Li, Z.; Tu, X.; Chen, Y.; Lin, W. HetDDI: a pre-trained heterogeneous graph neural network model for drug–drug interaction prediction. *Briefings in Bioinformatics* **2023**, *24*, bbad385.

[14] Saifuddin, K.M.; Bumgardner, B.; Tanvir, F.; Akbas, E. HyGNN: Drug-Drug Interaction Prediction via Hypergraph Neural Network. In Proceedings of the 2023 IEEE 39th International Conference on Data Engineering (ICDE), 2023, pp. 1503–1516. https://doi.org/10.1109/ICDE55515.2023.00119.

[15] Nguyen, D.A.; Nguyen, C.H.; Mamitsuka, H. Central-Smoothing Hypergraph Neural Networks for Predicting Drug–Drug Interactions. *IEEE Transactions on Neural Networks and Learning Systems* **2024**, *35*, 11620–11625. https://doi.org/10.1109/TNNLS.2023.3261860.

[16] Qiao, J.; Guo, X.; Jin, J.; Wang, D.; Li, K.; Gao, W.; Cui, F.; Zhang, Z.; Shi, H.; Wei, L. Taco-DDI: accurate prediction of drug-drug interaction events using graph transformer-based architecture and dynamic co-attention matrices. *Neural Networks* **2025**, *189*, 107655.

[17] Zhu, H.W.; Yi, H.C.; You, Z.H.; He, S.H.; Shang, X. A Multimodal Information Fusion Framework for Accurate and Robust Prediction of Drug-Drug Interactions. *Information Fusion* **2025**, p. 103981.

[18] Zheng, L.; Dai, B.; Yang, F.; Xia, J.; Lin, K.; Cai, Z. MFHNN-DDI: Molecular fragment-based hypergraph neural networks for drug–drug interaction prediction. *Neurocomputing* **2025**, p. 131958.

[19] Zhou, F.; Uddin, S. FG-DDI: Functional group-aware graph neural networks for drug-drug interaction prediction. *Journal of Biomedical Informatics* **2026**, p. 104981.

[20] Li, Y.; Liu, J.M.; Li, Y.C.; You, H.R.; You, Z.H.; Yuan, Y.; Mi, C.G. KA-DDI: A Knowledge-Adaptive Contrastive Learning Framework for Drug-Drug Interaction Prediction. *Expert Systems with Applications* **2026**, p. 131076.

[21] Li, D.; Yang, Y.; Cui, Z.; Yin, H.; Hu, P.; Hu, L. LLM-DDI: leveraging large language models for drug-drug interaction prediction on biomedical knowledge graph. *IEEE Journal of Biomedical and Health Informatics* **2025**.

[22] Ahmad, M.M.; Shah, Z.B.A.; Nies, H.W. A systematic review of molecular structures, knowledge graphs, and cold-start scenario in drug-drug interaction prediction. *Computers in Biology and Medicine* **2025**, *190*, 110122.

[23] Chen, Y.; Wang, J.; Zou, Q.; Niu, M.; Ding, Y.; Song, J.; Wang, Y. DrugDAGT: a dual-attention graph transformer with contrastive learning improves drug-drug interaction prediction. *BMC biology* **2024**, *22*, 233.

[24] Wang, Y.; Yang, Z.; Yao, Q. Accurate and interpretable drug-drug interaction prediction enabled by knowledge subgraph learning. *Communications Medicine* **2024**, *4*, 59.

[25] Tang, Z.; Chen, G.; Yang, H.; Zhong, W.; Chen, C.Y.C. DSIL-DDI: a domain-invariant substructure interaction learning for generalizable drug–drug interaction prediction. *IEEE Transactions on Neural Networks and Learning Systems* **2023**, *35*, 10552–10560.

[26] Li, Z.; Zhu, S.; Shao, B.; Zeng, X.; Wang, T.; Liu, T.Y. DSN-DDI: an accurate and generalized framework for drug–drug interaction prediction by dual-view representation learning. *Briefings in Bioinformatics* **2023**, *24*, bbac597.

[27] Wang, Y.; Ma, H.; Gao, Z.; Li, Z.; Chang, L. Criterion-guided polypharmacy side effects prediction with dual-view contrastive learning. *Applied Soft Computing* **2025**, p. 113814.

[28] Vo, T.H.; Nguyen, N.T.K.; Kha, Q.H.; Le, N.Q.K. On the road to explainable AI in drug-drug interactions prediction: A systematic review. *Computational and Structural Biotechnology Journal* **2022**, *20*, 2112–2123.

[29] Vaswani, A.; Shazeer, N.; Parmar, N.; Uszkoreit, J.; Jones, L.; Gomez, A.N.; Kaiser, L.; Polosukhin, I. Attention Is All You Need. In Proceedings of the Advances in Neural Information Processing Systems, 2017, Vol. 30.

[30] Yang, Z.; Zhong, W.; Lv, Q.; Chen, C.Y.C. Learning size-adaptive molecular substructures for explainable drug–drug interaction prediction by substructure-aware graph neural network. *Chemical science* **2022**, *13*, 8693–8703.

[31] Gan, Y.; Liu, W.; Xu, G.; Yan, C.; Zou, G. DMFDDI: deep multimodal fusion for drug–drug interaction prediction. *Briefings in Bioinformatics* **2023**, *24*, bbad397.

[32] Li, Y.; You, Z.H.; Yang, Y.; Mi, C.g. KGMAEDDI: Knowledge Graph and Molecular-Graph Masked Autoencoder for Drug-Drug Interaction Prediction. *IEEE Journal of Biomedical and Health Informatics* **2025**.

[33] Tran, B.H.; Hoang, H.M.; Nguyen, B.N.; Can, D.C.; Le, H.Q. A multifaceted approach to drug-drug interaction extraction with fusion strategies. *Journal of biomedical informatics* **2025**, p. 104874.

[34] Page, M.J.; McKenzie, J.E.; Bossuyt, P.M.; Boutron, I.; Hoffmann, T.C.; Mulrow, C.D.; Shamseer, L.; Tetzlaff, J.M.; Akl, E.A.; Brennan, S.E.; et al. The PRISMA 2020 statement: an updated guideline for reporting systematic reviews. *bmj* **2021**, *372*.

[35] Haddaway, N.R.; Page, M.J.; Pritchard, C.C.; McGuinness, L.A. PRISMA2020: An R package and Shiny app for producing PRISMA 2020-compliant flow diagrams, with interactivity for optimised digital transparency and Open Synthesis. *Campbell Systematic Reviews* **2022**, *18*, e1230. https://doi.org/10.1002/cl2.1230.

[36] Xia, Q.; Xiao, Z.; Liu, S.; Guo, J.; Zhou, Y.; Hu, W.; Lu, X. MOPDDI: Predicting drug–Drug interaction events based on multimodal mutual orthogonal projection and intermodal consistency loss. *IEEE Journal of Biomedical and Health Informatics* **2023**, *29*, 1782–1792.

[37] Chen, M.; Zhang, M.; Qu, C. Towards Interpretable Drug-Drug Interaction Prediction: A Graph-Based Approach with Molecular and Network-Level Explanations. In Proceedings of the Proceedings of the 31st ACM SIGKDD Conference on Knowledge Discovery and Data Mining V. 2, 2025, pp. 203–214.

[38] You, X.; Jia, Y.; Lu, L.; Yuan, Z. SynCaps-DDI: A hybrid neural model combining capsule networks and syntactic graph convolution for drug–drug interaction extraction. *Biomedical Signal Processing and Control* **2026**, *113*, 109230.

[39] He, Y.; Ma, T.; Li, C.; Ma, P.; Xiang, H.; Wang, J.; Liu, Y.; Song, B.; Zeng, X. ImageDDI: Image-enhanced molecular motif sequence representation for drug-drug interaction prediction. *Information Fusion* **2025**, p. 103574.

[40] Shen, X.; Gao, J.; Lyu, T.; Chen, J.; Zhang, J.; He, J.; Li, Z.; Yu, W. Enhancing drug-drug interaction event prediction from knowledge graphs by multimodal deep neural networks. *International Journal of Data Mining and Bioinformatics* **2025**, *29*, 338–361.

[41] Zhong, J.; Zhao, H.; Liang, X.; Zhao, Q.; Wang, J. MRLF-DDI: A Multi-view Representation Learning Framework for Drug-Drug Interaction Event Prediction. *IEEE Journal of Biomedical and Health Informatics* **2025**.

[42] Zhao, H.; Liu, Y.; Zhang, P.; Min, X.; Wang, M.; Che, C. MEDL-DDI: Example-Driven Learning With Multi-Source Features for Predicting Drug-Drug Interaction. *IEEE Journal of Biomedical and Health Informatics* **2025**.

[43] Zhao, C.; Han, D.; Zuo, Z.; Tohti, T. KGDB-DDI: Knowledge graph-based drug background data fusion model for drug-drug interaction prediction. *Artificial Intelligence in Medicine* **2025**, p. 103225.

[44] Zhang, Y.; Xu, X.; Feng, B.; Zheng, H.; Zhang, C.; Xu, W.; Deng, Z. RCAN-DDI: Relation-aware cross adversarial network for drug-drug interaction prediction. *Journal of Pharmaceutical Analysis* **2025**, *15*, 101159.

[45] Zhang, S.; Yang, Z.; Jin, S. AI-DDI: An Attention-Based Substructure Interactive Model for Predicting Drug-Drug Interaction. In Proceedings of the Proceedings of the 2025 5th International Conference on Bioinformatics and Intelligent Computing, 2025, pp. 396–402.

[46] Zhang, S.; Yu, C.; Zhang, C. SCATrans: semantic cross-attention transformer for drug–drug interaction predication through multimodal biomedical data. *BMC bioinformatics* **2025**, *26*, 157.

[47] Yan, X.; Gu, C.; Feng, Y.; Han, J. Predicting drug-drug interaction with graph mutual interaction attention mechanism. *Methods* **2024**, *223*, 16–25.

[48] Ma, M.; Lei, X. A dual graph neural network for drug–drug interactions prediction based on molecular structure and interactions. *PLOS Computational Biology* **2023**, *19*, e1010812.

[49] Yang, J.; Ding, Y.; Long, S.; Poon, J.; Han, S. DDI-MuG: Multi-Aspect Graphs for Drug-Drug Interaction Extraction. *Frontiers in Digital Health* **2023**, *5*, 1154133.

[50] Niu, D.; Xu, L.; Pan, S.; Xia, L.; Li, Z. SRR-DDI: a drug–drug interaction prediction model with substructure refined representation learning based on self-attention mechanism. *Knowledge-Based Systems* **2024**, *285*, 111337.

[51] Zhang, J.; Zhang, S.; Li, X.; Wu, D.; Wang, S.; Li, L.; Du, W.; Wang, Y. IIB-DDI: Invariant Information Bottleneck Theory for Out-of-Distribution Drug-Drug Interaction Prediction. *IEEE Transactions on Computational Biology and Bioinformatics* **2025**, *22*, 1009–1022.

[52] Yuan, Z.; Zhang, S.; Zhang, H.; Xie, P.; Jia, Y. Optimized drug-drug interaction extraction with BioGPT and focal loss-based attention. *IEEE Journal of Biomedical and Health Informatics* **2025**.

[53] Wang, J.; Li, R.; Zhang, S.; Xing, Y.; Yan, S.; Ma, L. A novel memory interaction neural network for multi-label drug-drug interaction prediction with neighbor importance sampling. *Artificial Intelligence in Medicine* **2025**, p. 103275.

[54] Wang, H.; Zhuang, L.; Ding, Y.; Tiwari, P.; Liang, C. EDDINet: Enhancing drug–drug interaction prediction via information flow and consensus constrained multi-graph contrastive learning. *Artificial Intelligence in Medicine* **2025**, *159*, 103029.

[55] Wang, G.; Chen, H.; Wang, H.; Gao, H.; Hu, X.; Cao, C. Mmddi-sse: A novel multi-modal feature fusion model with static subgraph embedding for drug-drug interaction event prediction. *IEEE Journal of Biomedical and Health Informatics* **2025**.

[56] Tamir, A.; Yuan, J.S. Kite-ddi: A knowledge graph integrated transformer model for accurately predicting drug-drug interaction events from drug smiles and biomedical knowledge graph. *IEEE Access* **2025**.

[57] Sun, J.; Zheng, H. HDN-DDI: a novel framework for predicting drug-drug interactions using hierarchical molecular graphs and enhanced dual-view representation learning. *BMC bioinformatics* **2025**, *26*, 28.

[58] Su, X.; Zhao, B.; Li, G.; Zhang, J.; Hu, P.; You, Z.; Hu, L. Knowledge graph neural network with spatial-aware capsule for drug-drug interaction prediction. *IEEE journal of biomedical and health informatics* **2024**, *29*, 1771–1781.

[59] Mashrafi, M.J.; Alif, H.A.; Uddin, M.; Selim, S.; Haldar, U.; Tabassum, M.B. Contrastive Hypergraph Networks with Adaptive Negative Sampling for Accurate Discovery and Risk Calibration of Low-Frequency Drug-Drug Interactions. In Proceedings of the 2025 International Conference on Intelligent Communication Networks and Computational Techniques (ICICNCT). IEEE, 2025, pp. 1–8. https://doi.org/10.1109/ICICNCT66124.2025.11232943.

[60] Luo, Y.; Deng, L.; Huang, Z. HLN-DDI: hierarchical molecular representation learning with co-attention mechanism for drug-drug interaction prediction. *BMC bioinformatics* **2025**, *26*, 152.

[61] Luo, T.; Lin, T.; Yang, C.; Fan, L.; Wang, W. A drug-drug interaction prediction method based on atomic 3d position encoding and elastic message passing graph neural network. *IEEE Journal of Biomedical and Health Informatics* **2025**.

[62] Liu, H.; Li, S.; Yu, Z. Predicting drug-drug interactions using heterogeneous graph neural networks: HGNN-DDI. *arXiv preprint arXiv:2508.18766* **2025**.

[63] Li, W.; Zhou, Y.; Ma, W.; Liang, W.; Tang, X. MDG-DDI: multi-feature drug graph for drug-drug interaction prediction. *BMC bioinformatics* **2025**, *26*, 268.

[64] Geng, G.; Wang, L.; Xu, Y.; Wang, T.; Ma, W.; Duan, H.; Zhang, J.; Mao, A. Mgddi: A multi-scale graph neural networks for drug–drug interaction prediction. *Methods* **2024**, *228*, 22–29.

[65] Li, D.; Zhao, F.; Yang, Y.; Cui, Z.; Hu, P.; Hu, L. Multi-view contrastive learning for drug-drug interaction event prediction. *IEEE journal of biomedical and health informatics* **2025**.

[66] Li, A.; Peng, J.; Wu, P. MMFF-DDI: An Efficient Multi-Modal Multi-Granularity Feature Fusion Framework for Drug-Drug Interaction Prediction. In Proceedings of the 2025 International Joint Conference on Neural Networks (IJCNN). IEEE, 2025, pp. 1–8.

[67] Jiang, M.; Liu, G.; Su, Y.; Jin, W.; Zhao, B. Hierarchical multi-relational graph representation learning for large-scale prediction of drug-drug interactions. *IEEE Transactions on Big Data* **2025**, *11*, 961–975.

[68] Li, Y.; Hou, L.X.; You, Z.H.; Yuan, Y.; Mi, C.G.; Huang, Y.a.; Yi, H.C. Mrgcddi: Multi-relation graph contrastive learning without data augmentation for drug-drug interaction events prediction. *IEEE Journal of Biomedical and Health Informatics* **2024**.

[69] Guo, L.; Lei, X.; Liu, L.; Chen, M.; Pan, Y. DualC: drug-drug interaction prediction based on dual latent feature extractions. *IEEE Transactions on Emerging Topics in Computational Intelligence* **2024**, *9*, 946–960.

[70] Ge, D.; Song, X.; Zhang, G.; Yan, J.; Li, J.; Liu, J.x. MSHGCL: Multi-Scale Hierarchical Graph Contrastive Learning for Drug-Drug Interactions. *IEEE Journal of Biomedical and Health Informatics* **2025**.

[71] Zhang, R.; Wang, X.; Wang, P.; Meng, Z.; Cui, W.; Zhou, Y. HTCL-DDI: a hierarchical triple-view contrastive learning framework for drug–drug interaction prediction. *Briefings in Bioinformatics* **2023**, *24*, bbad324.

[72] Deng, Z.; Xu, J.; Feng, Y.; Dong, L.; Zhang, Y. MAVGAE: a multimodal framework for predicting asymmetric drug–drug interactions based on variational graph autoencoder. *Computer Methods in Biomechanics and Biomedical Engineering* **2025**, *28*, 1098–1110.

[73] Cheng, Z.; Wang, Z.; Tang, X.; Hu, X.; Yang, F.; Yan, X. A Multi-View Feature-Based Interpretable Deep Learning Framework for Drug-Drug Interaction Prediction. *Interdisciplinary Sciences: Computational Life Sciences* **2025**, *17*, 437–448.

[74] Hu, B.; Yu, Z.; Li, M. Mphgcl-ddi: meta-path-based heterogeneous graph contrastive learning for drug-drug interaction prediction. *Molecules* **2024**, *29*, 2483.

[75] Zhu, J.; Che, C.; Jiang, H.; Xu, J.; Yin, J.; Zhong, Z. SSF-DDI: a deep learning method utilizing drug sequence and substructure features for drug–drug interaction prediction. *BMC bioinformatics* **2024**, *25*, 39.

[76] Zhong, J.; Zhao, H.; Zhao, Q.; Wang, J. A knowledge graph-based method for drug-drug interaction prediction with contrastive learning. *IEEE/ACM Transactions on Computational Biology and Bioinformatics* **2024**, *21*, 2485–2495.

[77] Chen, J.; Deng, Q.; Zhen, P.; Hu, J.; Wang, Y.; Peng, J.; You, Z.; Shang, X.; Zhang, X.; Wang, T. Knowledge Graph-Enhanced Multi-Scale Modeling for Drug-Drug Interaction Prediction. *Molecular Therapy Nucleic Acids* **2026**.

[78] Jeong, Y.U.; Choi, J.; Park, N.; Ryu, J.Y.; Kim, Y.R. Predicting drug-drug interactions: a deep learning approach with GCN-based collaborative filtering. *Artificial Intelligence in Medicine* **2025**, *167*, 103185.

[79] Gong, L.; Wei, X.; Ji, H.; Huang, H.; Ji, Y. A Graph-based Multi-dimensional Interaction Network for Drug-Drug Interaction Prediction. *IEEE Journal of Biomedical and Health Informatics* **2025**.

[80] Aromataris, E.; Pearson, A. The systematic review: an overview. *AJN The American Journal of Nursing* **2014**, *114*, 53–58.

[81] Gao, J.; Wu, Z.; Al-Sabri, R.; Oloulade, B.M.; Chen, J. Autoddi: drug–drug interaction prediction with automated graph neural network. *IEEE Journal of Biomedical and Health Informatics* **2024**, *28*, 1773–1784.

[82] Wang, J.; Du, H.; Li, Y. DDI-AttendNet: cross attention with structured graph learning for inter-drug connectivity analysis. *Frontiers in Pharmacology* **2025**, *16*, 1680655.

[83] Tanvir, F.; Saifuddin, K.M.; Islam, M.I.K.; Akbas, E. DDI Prediction With Heterogeneous Information Network - Meta-Path Based Approach. *IEEE/ACM Transactions on Computational Biology and Bioinformatics* **2024**, *21*, 1168–1179. https://doi.org/10.1109/TCBB.2024.3417715.

[84] Kundi, I.N.; Sheikh, S.A.; Malik, F.M.; Bhatti, K.A. DDI-KGAT: A Graph Attention Network on Biomedical Knowledge Graph for the Prediction of Drug-Drug Interactions. *IEEE Access* **2024**, *12*, 162028–162039.

[85] Liu, S.; Zhang, Y.; Cui, Y.; Qiu, Y.; Deng, Y.; Zhang, Z.; Zhang, W. Enhancing drug-drug interaction prediction using deep attention neural networks. *IEEE/ACM transactions on computational biology and Bioinformatics* **2022**, *20*, 976–985.

[86] Huang, L.; Chen, Q.; Lan, W. Predicting drug–drug interactions based on multi-view and multichannel attention deep learning. *Health Information Science and Systems* **2023**, *11*, 50.

[87] Chen, Y.; Ma, T.; Yang, X.; Wang, J.; Song, B.; Zeng, X. MUFFIN: multi-scale feature fusion for drug–drug interaction prediction. *Bioinformatics* **2021**, *37*, 2651–2658.

[88] Xu, C.; Bulusu, K.C.; Pan, H.; Elemento, O. Ddi-gpt: Explainable prediction of drug-drug interactions using large language models enhanced with knowledge graphs. *BioRxiv* **2024**, p. 2024.12.06.627266. https://doi.org/10.1101/2024.12.06.627266.

[89] Feng, Q.; Huang, X. Multi-feature machine learning for enhanced drug–drug interaction prediction. *Journal of Biomedical Informatics* **2025**, *171*, 104923.

[90] Gallifant, J.; Afshar, M.; Ameen, S.; Aphinyanaphongs, Y.; Chen, S.; Cacciamani, G.; Demner-Fushman, D.; Dligach, D.; Daneshjou, R.; Fernandes, C.; et al. The TRIPOD-LLM reporting guideline for studies using large language models. *Nature Medicine* **2025**, *31*, 60–69. https://doi.org/10.1038/s41591-024-03425-5.

[91] Zheng, W.; Li, Z.; Chen, Y.; Liao, W.; Deng, L.; Zhang, H.; Lin, Y.; Peng, Y. GEP-DNN4Mol: Automatic chemical molecular design based on deep neural networks and gene expression programming. *Health Information Science and Systems* **2025**, *13*, 31. https://doi.org/10.1007/s13755-025-00344-8.

[92] Hu, X.; Lu, Y.; Tian, G.; Bing, P.; Wang, B.; He, B. Predicting herb-disease associations through graph convolutional network. *Current Bioinformatics* **2023**, *18*, 610–619. https://doi.org/10.2174/1574893618666230504143647.

[93] Tang, X.; Cai, L.; Meng, Y.; Xu, J.; Lu, C.; Yang, J. Indicator regularized non-negative matrix factorization method-based drug repurposing for COVID-19. *Frontiers in Immunology* **2021**, *11*, 603615. https://doi.org/10.3389/fimmu.2020.603615.

[94] Hong, Y.; Luo, P.; Jin, S.; Liu, X. LaGAT: link-aware graph attention network for drug–drug interaction prediction. *Bioinformatics* **2022**, *38*, 5406–5412.

[95] Qian, Y.; Wang, Q.; Yin, L.; Lu, A.Y. CF-DTI: Coarse-to-fine feature extraction for enhanced drug-target interaction prediction. *Health Information Science and Systems* **2025**, *13*, 55. https://doi.org/10.1007/s13755-025-00370-6.

[96] Chandak, P.; Huang, K.; Zitnik, M. Building a knowledge graph to enable precision medicine. *Scientific data* **2023**, *10*, 67.

[97] Zhou, L.; Li, Z.; Yang, J.; Tian, G.; Liu, F.; Wen, H.; Peng, L.; Chen, M.; Xiang, J.; Peng, L. Revealing drug-target interactions with computational models and algorithms. *Molecules* **2019**, *24*, 1714. https://doi.org/10.3390/molecules24091714.

[98] Huang, C.; Wang, Y.; Jiang, Y.; Li, M.; Huang, X.; Wang, S.; Pan, S.; Zhou, C. Flow2GNN: Flexible two-way flow message passing for enhancing GNNs beyond homophily. *IEEE Transactions on Cybernetics* **2024**, *54*, 6607–6618. https://doi.org/10.1109/TCYB.2024.3412149.

[99] Meng, Y.; Wang, Y.; Xu, J.; Lu, C.; Tang, X.; Peng, T.; Zhang, B.; Tian, G.; Yang, J. Drug repositioning based on weighted local information augmented graph neural network. *Briefings in Bioinformatics* **2024**, *25*, bbad431. https://doi.org/10.1093/bib/bbad431.

[100] Xiang, J.; Zhang, N.R.; Zhang, J.S.; Lv, X.Y.; Li, M. PrGeFNE: Predicting disease-related genes by fast network embedding. *Methods* **2021**, *192*, 3–12. https://doi.org/10.1016/j.ymeth.2020.06.015.

[101] Wang, S.; Yang, C.; Chen, L. LSA-DDI: Learning stereochemistry-aware drug interactions via 3D feature fusion and contrastive cross-attention. *International Journal of Molecular Sciences* **2025**, *26*, 6799. https://doi.org/10.3390/ijms26146799.

[102] Tian, Y.; Yi, J.; Wang, N.; Wu, C.; Peng, J.; Liu, S.; Yang, G.; Cao, D. DDInter 2.0: An enhanced drug interaction resource with expanded data coverage, new interaction types, and improved user interface. *Nucleic Acids Research* **2025**, *53*, D1356–D1362. https://doi.org/10.1093/nar/gkae726.

[103] University of Liverpool. Drug–Drug Interaction Resources, 2026. Accessed on 30 July 2026.

[104] Medscape. Drug Interactions Checker, 2026. Accessed on 30 July 2026.

[105] DrugBank. Drug–Drug Interaction Checker, 2026. Accessed on 30 July 2026.

[106] Wolters Kluwer. UpToDate Lexidrug: Drug Decision Support for Health Professionals, 2026. Accessed on 30 July 2026.

[107] Merative. Micromedex Drug Database, 2026. Accessed on 30 July 2026.

[108] Zhong, Y.; Zheng, H.; Chen, X.; Zhao, Y.; Gao, T.; Dong, H.; Luo, H.; Weng, Z. DDI-GCN: Drug–drug interaction prediction via explainable graph convolutional networks. *Artificial Intelligence in Medicine* **2023**, *144*, 102640. https://doi.org/10.1016/j.artmed.2023.102640.

[109] Dmitriev, A.; Filimonov, D.; Lagunin, A.; Karasev, D.; Pogodin, P.; Rudik, A.; Poroikov, V. Prediction of Severity of Drug–Drug Interactions Caused by Enzyme Inhibition and Activation. *Molecules* **2019**, *24*, 3955. https://doi.org/10.3390/molecules24213955.

[110] Yu, H.; Li, K.; Dong, W.; Song, S.; Gao, C.; Shi, J. Attention-based cross domain graph neural network for prediction of drug–drug interactions. *Briefings in Bioinformatics* **2023**, *24*, bbad155.

[111] Wu, D.; Sun, W.; He, Y.; Chen, Z.; Luo, X. MKG-FENN: A multimodal knowledge graph fused end-to-end neural network for accurate drug–drug interaction prediction. In *Proceedings of the AAAI Conference on Artificial Intelligence*; 2024; Volume 38, pp. 10216–10224.

[112] Jiang, Z.; Gong, Z.; Dai, X.; Zhang, H.; Ding, P.; Shen, C. Deep graph contrastive learning model for drug–drug interaction prediction. *PLOS ONE* **2024**, *19*, e0304798.
